# Epidemiology of children and adolescents undergoing surgery at Mexican public hospitals: a retrospective registry-based analysis from 2010 to 2022

**DOI:** 10.1186/s12887-026-07011-1

**Published:** 2026-05-21

**Authors:** Magdalena Gruendl, Letícia Nunes Campos, Tanujit Dey, Theoneste Nkurunziza, Taylor Wurdeman, Gunther Schauberger, Arturo Cervantes Trejo, Jaime Shalkow-Klincovstein, Stefanie J. Klug, Tarsicio Uribe-Leitz

**Affiliations:** 1 Chair of Epidemiology, TUM School of Medicine and Health, Technical University of Munich, Munich, Germany; 2 Program in Global Surgery and Social Change, Harvard Medical School, Boston, USA; 3 Faculty of Medical Sciences, Federal University of Pernambuco, Recife, Brazil; 4 The Center for Surgery and Public Health, Brigham and Women’s Hospital, Boston, USA; 5 Faculty of Health Sciences, Anahuac University, Anahuac, Mexico; 6 Pediatric Surgical Oncology, ABC Medical Center, Mexico City, Mexico

**Keywords:** Pediatric surgery, Children and adolescents, Mexico, Epidemiological data analysis, Surgical epidemiology

## Abstract

**Introduction:**

Surgical care is essential for addressing acute and chronic conditions in children and adolescents. While national-level data on pediatric surgical procedures in Mexico exist, analysis of such data remain limited. This study aimed to describe geographic variations and temporal trends in pediatric surgical procedures performed at public hospitals from 2010 to 2022, including the impact of the COVID-19 pandemic, and to identify factors associated with in-hospital mortality.

**Methods:**

This retrospective registry-based analysis used hospital discharge data from Mexico’s Ministry of Health (MoH) public hospitals (2010–2022), including all elective and emergency surgical procedures in patients aged 0–17. Descriptive statistics summarized demographic and clinical characteristics. Surgical specialties stratified by age and sex were visualized using sankey flow diagrams. Age-standardized incidence rates (ASIR) were calculated per 100,000 children and adolescents by state and year using the WHO world standard population. Interrupted time series (ITS) analysis with Poisson regression evaluated trends of surgical procedure volume. Logistic regression identified factors associated with in-hospital mortality.

**Results:**

Among 752,654 pediatric surgical patients, 58.2% were male and 41.8% female. The most common age group was 10–14 years (27.7%). Overall, 42.4% were normal weight, 16.6% of patients were underweight, 21.6% overweight, 19.4% obese. Additionally, 2.1% identified as indigenous. General surgery (46.8%) and orthopedic surgery (23.4%) were the most frequent surgical procedures. The ASIR of surgical procedures was 141.8 per 100,000 children and adolescents nationwide (2010–2022). Guanajuato reported the highest overall ASIR (333.8 per 100,000), while Nuevo León had the lowest (18.5 per 100,000). Surgical procedure volumes increased until 2015, declined thereafter, and dropped sharply by 35.0% in April 2020 at the onset of the nationwide lockdown (exp(β): 0.65, 95% CI: 0.59–0.70), with volumes gradually recovering by 2022. Hospital-acquired infections (HAIs) (aOR: 2.71, 95% CI: 2.46–2.98) and prolonged length of stay (LOS) (aOR: 2.27, 95% CI: 2.13–2.41) were associated with increased in-hospital mortality.

**Conclusion:**

This national analysis demonstrates pronounced geographic and temporal disparities in pediatric surgical care across Mexico’s public hospitals, including substantial declines during the COVID-19 pandemic. Coordinated investments in pediatric surgical infrastructure, state-level health information systems, and referral networks are critical to ensuring equitable, evidence-based pediatric surgical services.

**Supplementary Information:**

The online version contains supplementary material available at 10.1186/s12887-026-07011-1.

## Introduction

Surgical care is an essential component of healthcare for children and adolescents, enabling the treatment of a broad range of acute and chronic conditions [1, 2]. Timely access to safe and effective surgical services is critical for reducing preventable morbidity and mortality in this population [3]. However, an estimated 1.7 billion children globally lack access to the basic, life-saving surgical care they require [4]. In many low- and middle-income countries (LMICs), pediatric surgical services remain under-resourced and insufficiently monitored, limiting efforts to evaluate and improve service delivery [5, 6]. In Central Latin America, pediatric surgical capacity remains critically limited, with only 35% of children having access to surgical care within 2 h [7]. Although global surgery has gained increasing attention, pediatric surgical care continues to be underrepresented in policy discussions and investment priorities [8]. Addressing these inequities is central to the United Nations Sustainable Development Goals (SDG), particularly SDG 3 (Good Health and Well-being) and SDG 10 (Reduced Inequalities), which emphasize equitable access to essential health services for children [9].

In Mexico, an upper middle-income country, challenges in pediatric surgical care are compounded by structural barriers rooted in a fragmented healthcare system characterized by institutional segmentation and substantial disparities in access to care [10]. Mexico’s healthcare system consists of multiple public and private subsystems that serve different population groups based primarily on employment and insurance status. This structure has led to unequal resource allocation, fragmented service delivery, and regional variability in care quality [11, 12]. Approximately 3–6% of the population solely relies on private healthcare services [13], while the vast majority depends on publicly funded institutions, primarily supported by the federal government. In 2022, an estimated 50.4 million Mexicans (39.1% of the population) lacked access to health services [13, 14].

Public hospitals administered by the Ministry of Health (Secretaría de Salud, MoH) are the primary providers of surgical services for children and adolescents whose families lack private insurance or employment-based social security [15, 16]. These MoH facilities are part of Mexico’s public hospital network, which accounts for about 30% of the country’s hospitals [13, 17]. Patients treated in these facilities were insured under Seguro Popular (Mexico’s Universal Health Coverage program) until its replacement by the Instituto de Salud para el Bienestar (Institute of Health for Well-being, INSABI) in late 2019 and early 2020, as part of a broader reform in Mexico’s public universal healthcare coverage policy [13]. INSABI was subsequently dissolved and merged into Instituto Mexicano del Seguro Social-Bienestar (Mexican Social Security Institute for Well-being, IMSS-Bienestar) in 2023 [13, 18–20]. Under Seguro Popular, families paid modest, income-based annual contributions, with the poorest households fully exempt. In contrast, INSABI offered care free of charge without formal enrollment, and the current IMSS-Bienestar is formally affiliating and credentialing the population it serves, providing care at no cost at the point of service [13, 21–23]. In Mexico, pediatric surgery is a formally recognized medical specialty with dedicated residency training programs [24]. However, pediatric surgical care is not uniformly restricted to pediatric surgeons, particularly in secondary-level hospitals and resource-limited settings, where general surgeons may provide surgical care to children and adolescents. As a result, pediatric surgical services are delivered through a mixed-provider model within the public health system [25].

Children and adolescents account for approximately 30% of Mexico’s population [26], making it imperative to understand how they interact with the public healthcare system, particularly in surgical settings. While previous studies have examined pediatric surgical outcomes in select Mexican institutions or specialties [27, 28], no comprehensive, national analysis of pediatric surgical volume, service delivery, and outcomes, including in-hospital mortality, exists. Within this fragmented system, pediatric surgical specialists are present in Mexico but remain limited, with an estimated workforce density of 3.7 pediatric surgeons per 100,000 children [29]. Pediatric surgery training is highly centralized, with only 14 accredited pediatric surgery programs nationwide, seven located in Mexico City and the remainder in a small number of large urban centers, concentrating pediatric expertise in tertiary hospitals rather than secondary or rural facilities. National evaluations further describe an uneven distribution of surgical and anesthesia providers and report that only 82% of the population lives within two hours of a facility capable of providing essential surgical care, with clusters of underserved municipalities predominantly outside major urban hubs [30]. Significant regional disparities in capacity, infrastructure, and access to specialized care further complicate efforts to evaluate system performance, address inequities, and guide evidence-based policy [30]. The COVID-19 pandemic further disrupted surgical services through widespread delays, cancellations, and capacity shifts [31–33], but its specific impact on pediatric surgery within Mexico’s MoH-administered hospitals remains unexamined.

This study addresses these gaps through a retrospective, registry-based analysis of pediatric surgical procedures performed in MoH-administered public hospitals across Mexico from 2010 to 2022. Our objectives were to: (1) quantify the pediatric surgical burden across Mexican states, (2) analyze temporal trends, including the impact of the COVID-19 pandemic, and (3) identify factors associated with in-hospital mortality. Based on these objectives, we hypothesized that pediatric surgical procedure rates would vary substantially across Mexican states, reflecting inequities in access to care; that pediatric surgical volumes would undergo temporal changes and experience disruption during the COVID-19 pandemic, with heterogeneous effects across surgical specialties; and that patient- and system-level characteristics would be associated with in-hospital mortality.

## Methods

### Study design and data source

This retrospective, registry-based study analyzed all elective and emergency pediatric surgical procedures performed in MoH-administered public hospitals across Mexico between January 1, 2010, and December 31, 2022. Data were obtained from the automated hospital discharge database (Subsistema Automatizado de Egresos Hospitalarios, SAEH) maintained by the MoH [34]. SAEH is a national registry of de-identified hospitalization records and is publicly accessible via Cubos dinámicos [34], an open-access multidimensional analytical tool.

Population estimates used for incidence rate calculations were sourced from the National Institute for Statistics and Geography (Instituto Nacional de Estadística y Geografía, INEGI) [35]. April 2020 marked the start of the COVID-19 pandemic period in this analysis, following Mexico’s declaration of a national sanitary emergency and nationwide lockdown on March 30, 2020 [36].

### Study variables

Variables analyzed included sex (male, female), age category (under 5 years, 5–9 years, 10–14 years, 15–17 years) and calendar year (2010–2022). BMI-for-age category was assessed using body mass index (BMI) for age and sex, with z-scores derived from the World Health Organization (WHO) anthropometric reference standards [37]. These z-scores indicate how many standard deviations (SD) a child’s BMI deviates from the median BMI of an age- and sex-matched healthy reference population [37]. Classification followed WHO criteria: underweight (<-2 SD), normal weight (-2 to ≤ + 1 SD), overweight ( > + 1 to ≤ + 2 SD), and obese ( > + 2 SD) [37, 38]. Indigenous identity (no, yes) was defined based on self-reported indigenous status and/or the reporting of an indigenous language spoken, as recorded in the Ministry of Health hospital discharge database, consistent with national administrative data collection practices in Mexico [39]. Ward type (intensive care unit (ICU), internal medicine, pediatrics, surgery, other, missing) was also included. The category “other” includes patients admitted to wards that do not fit any of the predefined categories. In the SAEH registry, ward type reflects the hospital service recorded at admission in the administrative database. Surgical procedures (cardiothoracic surgery, endocrine surgery, general surgery, neurosurgery, multiple procedures, ophthalmology, orthopedic surgery, otolaryngology and urology) were classified using ICD-9-CM (International Classification of Diseases, 9th Revision, Clinical Modification) Volume 3 codes [40] and grouped into clinically meaningful categories based on AHRQ classifications (Agency for Healthcare Research and Quality) [41], with procedures aggregated into specialty-based categories rather than analyzed at the level of individual operative techniques or procedural complexity. The “multiple procedures” category included patients undergoing more than one procedure during the same hospital admission. Additional variables included type of anesthesia (combined, general, local, regional, sedation, missing), hospital-acquired infections (HAI; no, yes), length of stay (LOS; <4 days, ≥ 4 days), in-hospital mortality (no, yes), as well as state and number of surgical procedures (continuous), representing the surgical procedure volume.

HAI was defined as a nosocomial infection not present at the time of admission and acquired during hospitalization [42]. Prolonged LOS was defined as a hospital stay of 4 days or more (≥ 4 days), corresponding to the 75th percentile of the LOS distribution in the analysis population, a percentile-based threshold consistent with definitions used in previous pediatric surgical studies [43–45]. In-hospital mortality was defined as death occurring during hospitalization [46].

### Analysis population

We included eligible children and adolescents (aged 0–17 years) who underwent a surgical procedure at MoH-administered public hospitals in Mexico from 2010 to 2022, as recorded in the national hospital discharge database (SAEH). First-time admissions were included, while data from subsequent admissions of the same children and adolescents during the study period were excluded to avoid double-counting procedures and eliminate interpatient correlation. All surgical specialties were included in the analysis except obstetrical and gynecological procedures, which were excluded due to their distinct care pathways and limited comparability to other pediatric surgical services.

During data cleaning, we removed observations with missing values for sociodemographic variables (age, sex, indigenous identity) and anthropometric indicators to ensure valid classification of BMI-for-age category. Children and adolescents with missing BMI and BMI-for-age z-scores below − 7 or above + 7 SDs were excluded [47–49]. Among the 1,236,989 records eligible for sociodemographic and anthropometric assessment, indigenous identity was missing in 49,129 records (4.0%). Among records eligible for BMI-for-age calculation, BMI-for-age data were missing in 419,152 records (33.9%), and an additional 65,183 records (5.3%) were excluded due to implausible BMI-for-age z-scores. We conducted a supplementary analysis comparing in-hospital mortality between patients included in the analysis population and those excluded due to missing BMI-for-age data. We also excluded unspecified surgical procedures. For hospital-based variables (ward type and type of anesthesia), missing values were retained for descriptive analyses, incidence rate calculations, and trend analyses because differences might reflect facility-level documentation rather than patient characteristics. Supplementary Table 1 provides an overview of the inclusion and exclusion steps applied to the initially available 2,360,629 registry records. The final analysis population consisted of 752,654 records of children and adolescent.

### Statistical analysis

Descriptive statistics summarized demographic and clinical characteristics. Categorical variables were presented as frequencies and percentages. Surgical specialties stratified by age and sex were visualized using a Sankey flow diagram.

Age-standardized incidence rates (ASIR) were calculated per 100,000 children and adolescents (aged 0–17 years) by state and year using the WHO world standard population [50], based on pediatric surgical procedures recorded in Ministry of Health-administered hospitals; therefore, these rates reflect pediatric surgical utilization within the MoH hospital system rather than population-level surgical incidence. Shapefiles of Mexico’s first-level administrative divisions were obtained from the *Natural Earth* database version 5.1 [51].

We used interrupted time series (ITS) analysis with Poisson regression models to evaluate long-term trends and the impact of the COVID-19 pandemic. April 2020 was defined as the interruption point in the model, as it marked the first month following the nationwide implementation of COVID-19-related restrictions in Mexico, March 2020 was treated as a transition month and included in the pre-pandemic period, which is consistent with previous COVID-19 studies from Mexico [52, 53]. Model coefficients were estimated on the log scale, and exponentiated values were presented as multiplicative effects on the absolute number of surgeries with 95% confidence intervals (95% CIs). Seasonality was addressed by including month-of-year indicator variables in all ITS models, and autocorrelation was assessed using residual correlograms; robust standard errors were used throughout, and conclusions were driven by the pronounced April 2020 level change. To describe trends over time and facilitate interpretation, we calculated the average monthly number of surgical procedures per calendar year, both overall and by specialty, and reported the percent change in these averages.

First univariable and then multivariable logistic regression analyses were performed to determine factors associated with in-hospital mortality. The following confounders were considered in multivariable logistic regression due to their clinical relevance: sex, age category, BMI-for-age category, indigenous identity, ward type, surgical specialty, anesthesia type, HAI and LOS. For the multivariable logistic regression, we conducted a complete case analysis for ward type and anesthesia type, excluding records with missing values to ensure complete covariate information [54, 55]. Odds ratios (OR) and adjusted odds ratios (aOR) with 95% CIs were reported.

All statistical analysis were performed using STATA, version 18 (StataCorp, College Station, TX, USA). Visualization of the heatmaps and heat matrix was conducted in R, version 4.5.1 (R Foundation for Statistical Computing, Vienna, Austria).

This study was approved as exempt by the Harvard Faculty of Medicine IRB (protocol #IRB23-0178) based on use of de-identified, publicly available secondary data.

## Results

### Demographic and clinical characteristics

Among 752,654 pediatric surgical patients admitted between 2010 and 2022 to Mexican MoH hospitals, 58.2% were male and 41.8% were female (Table 1). The most common age group was 10–14 years (27.7%), followed by children under 5 years (26.0%), 15–17 years (23.8%), and 5–9 years (22.4%). BMI-for-age category was normal in 42.4% of children and adolescents, while 16.6% were underweight, 21.6% overweight, and 19.4% obese. Additionally, 2.1% of children and adolescents reported an indigenous identity. Most children and adolescents were treated in surgical (50.7%) or pediatric wards (21.5%), with only 0.1% admitted to the ICU. General surgery was the most frequently performed surgical procedure overall (46.8%), followed by orthopedic surgery (23.4%). Among male patients, general surgery accounted for 43.8% and orthopedic surgery for 27.7% of procedures. Among female patients, general surgery accounted for 51.1% of procedures and orthopedic surgery for 17.4%. General anesthesia was administered in 42.8% of patients, regional in 35.9%, local in 9.0%, sedation in 4.7%, and combined in 4.3%, with 3.3% missing data. HAIs were reported in 0.9% of patients, and 28.6% had a prolonged LOS. In-hospital mortality was 1.0% in both sexes. Overall in-hospital mortality among patients excluded from the analysis population due to missing BMI-for-age z-scores was 1.0%, identical to the mortality observed in the analysis population (Supplementary Table 2).

**Table 1:** Demographics and clinical characteristics of 752,654 children and adolescents stratified by sex in Mexico from 2010–2022

	Male		Female		Total	
	*n*	%	*n*	%	*n*	%
Age category						
Under 5 years	122,698	28.0	73,108	23.2	195,806	26.0
5–9 years	106,835	24.4	61,724	19.6	168,559	22.4
10–14 years	131,313	30.0	77,513	24.6	208,826	27.7
15–17 years	77,042	17.6	102,421	32.5	179,463	23.8
BMI-for-age category						
Underweight	77,887	17.8	47,326	15.0	125,213	16.6
Normal weight	182,745	41.7	136,308	43.3	319,053	42.4
Overweight	87,021	19.9	75,295	23.9	162,316	21.6
Obese	90,235	20.6	55,837	17.7	146,072	19.4
Indigenous identity						
No	429,567	98.1	307,391	97.7	736,958	97.9
Yes	8,321	1.9	7,375	2.3	15,696	2.1
Ward type						
ICU	269	0.1	182	0.1	451	0.1
Internal medicine	3,036	0.7	2,141	0.7	5,177	0.7
Pediatrics	87,699	20.0	74,436	23.6	162,135	21.5
Surgery	216,694	49.5	164,823	52.4	381,517	50.7
Other	105,823	24.2	57,298	18.2	163,121	21.7
Missing	24,367	5.6	15,886	5.0	40,253	5.3
Surgical procedure						
Cardiothoracic Surgery	11,535	2.6	9,279	2.9	20,814	2.8
Endocrine Surgery	733	0.2	943	0.3	1,676	0.2
General Surgery	191,743	43.8	160,692	51.1	352,435	46.8
Multiple procedures	17,315	4.0	51,119	16.2	68,434	9.1
Neurosurgery	9,902	2.3	6,810	2.2	16,712	2.2
Ophthalmology	6,760	1.5	6,427	2.0	13,187	1.8
Orthopedic Surgery	121,168	27.7	54,733	17.4	175,901	23.4
Otolaryngology	29,182	6.7	22,440	7.1	51,622	6.9
Urology	49,550	11.3	2,323	0.7	51,873	6.9
Type of anesthesia						
Combined	21,237	4.8	11,481	3.6	32,718	4.3
General	201,881	46.1	120,233	38.2	322,114	42.8
Local	24,441	5.6	43,214	13.7	67,655	9.0
Regional	153,845	35.1	116,416	37.0	270,261	35.9
Sedation	22,496	5.1	12,927	4.1	35,423	4.7
Missing	13,988	3.2	10,495	3.3	24,483	3.3
HAI						
No	433,922	99.1	311,664	99.0	745,586	99.1
Yes	3,966	0.9	3,102	1.0	7,068	0.9
LOS						
< 4 days	305,443	69.8	231,668	73.6	537,111	71.4
>= 4 days	132,445	30.2	83,098	26.4	215,543	28.6
In-hospital mortality						
No	433,638	99.0	311,554	99.0	745,192	99.0
Yes	4,250	1.0	3,212	1.0	7,462	1.0
Total	437,888	58.2	314,766	41.8	752,654	100.0

*n* Number of patients, *%* Percentage

*BMI* Body mass index, *Ward* Ward at admission; transfers during hospitalization are not captured, *ICU* Intensive care unit, *HAI* Hospital-acquired infection, *LOS* Length of stay

**Fig. 1:**
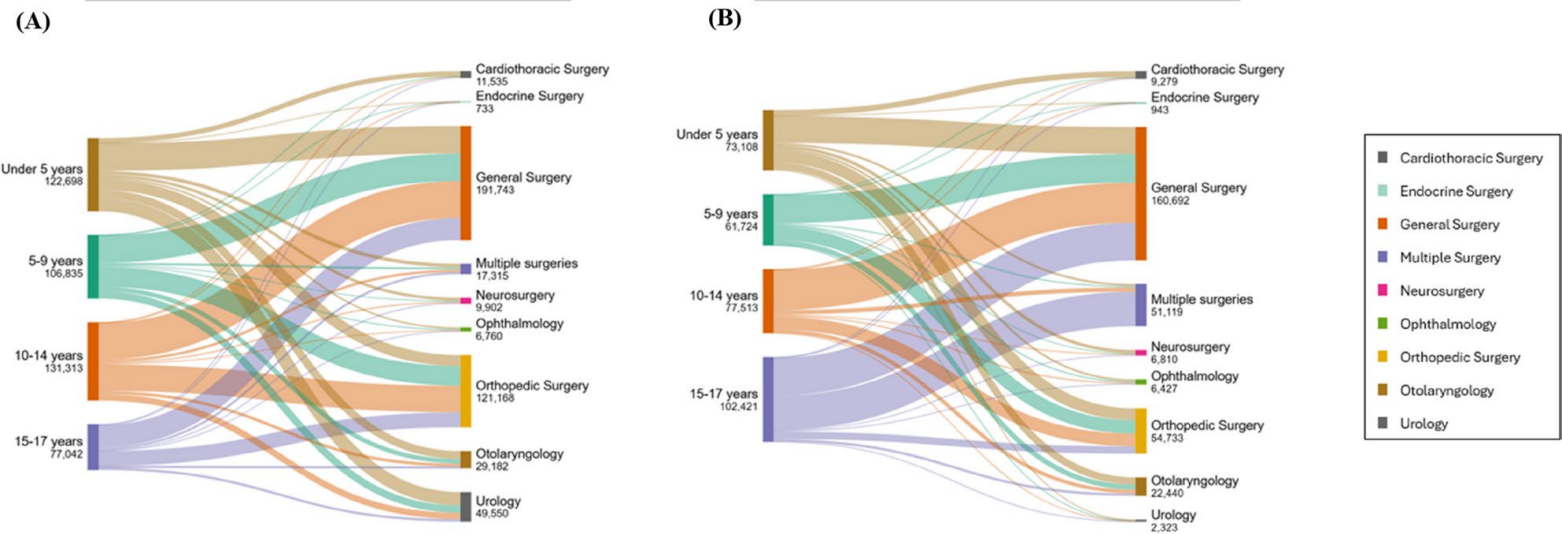
Surgical procedures (n=752,654) in Mexico, 2010-2022, stratified by sex, age, and surgical specialty. **A **Surgical procedures by age and specialty among males (n=437, 888). **B **Surgical procedures by age and specialty among females (n=314, 766)

The highest number of general surgery procedures among males (*n* = 191,743) was observed in those aged 10–14 years (*n* = 61,876), representing 32.3% of all male general surgeries (Fig. 1 and Supplementary Table 3). General surgery procedures among females (*n* = 160,692) were most frequent in adolescents aged 15–17 years (*n* = 45,451, 28.3%) (Fig. 1 and Supplementary Table 4). Among male patients, a substantial proportion of the 11,535 cardiothoracic surgeries were performed in children under 5 years (8,392 procedures; 72.8%). Similarly, among female patients, most cardiothoracic surgeries were also concentrated in this age group, with 7,040 of 9,279 procedures (75.9%) performed in children under 5 years.

### Incidence rates and geographic variations

**Fig. 2:**
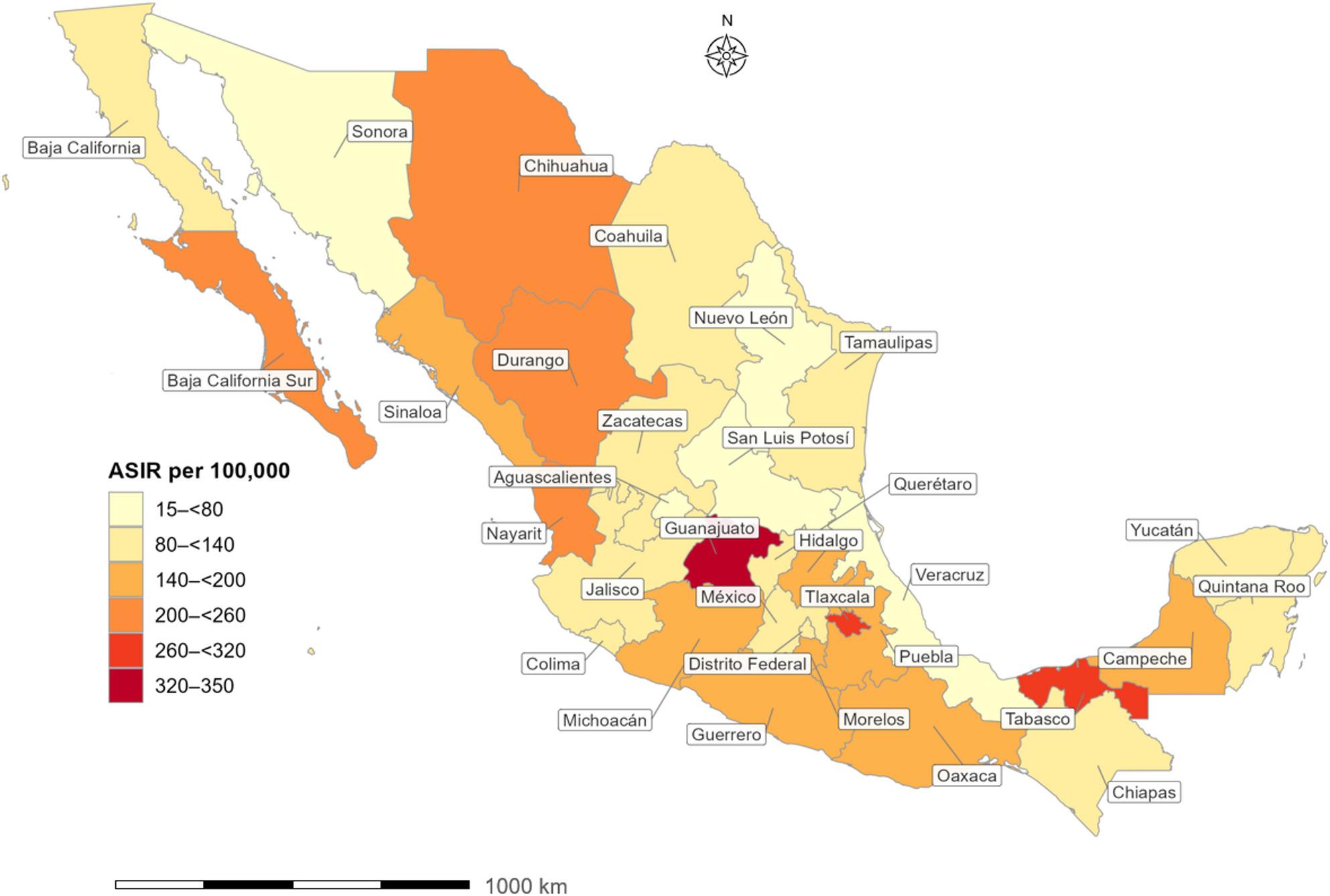
ASIR of surgical procedures per 100,000 children and adolescents in Mexico stratified by state from 2010-2022 (n=752,654). The map displays age-standardized incidence rates (ASIR) of surgical procedures performed in Ministry of Health (MoH)-administered hospitals per 100,000 children and adolescents across Mexico’s 32 federal entities. A red color gradient denotes increasing ASIR, with darker shades indicating higher procedure rates. The scale ranges from 18.5 to 333.8 cases per 100,000 population aged under 18 years and reflect pediatric surgical utilization within the MoH public hospital system, rather than population-level surgical incidence across all healthcare sectors

The overall ASIR of surgical procedures was 141.8 per 100,000 children and adolescents across all Mexican states and years (2010–2022) (Fig. 2 and Supplementary Table 5). These rates reflect surgical utilization within Ministry of Health–administered hospitals rather than true population-level surgical incidence, as procedures performed in other health system subsystems are not captured in this registry. Guanajuato reported the highest overall ASIR during this period (333.8 per 100,000), followed by Tabasco (319.3) and Tlaxcala (315.9). In contrast, consistently low overall rates were observed in Nuevo León (18.5), Aguascalientes (41.4), and Sonora (53.2).

**Fig. 3:**
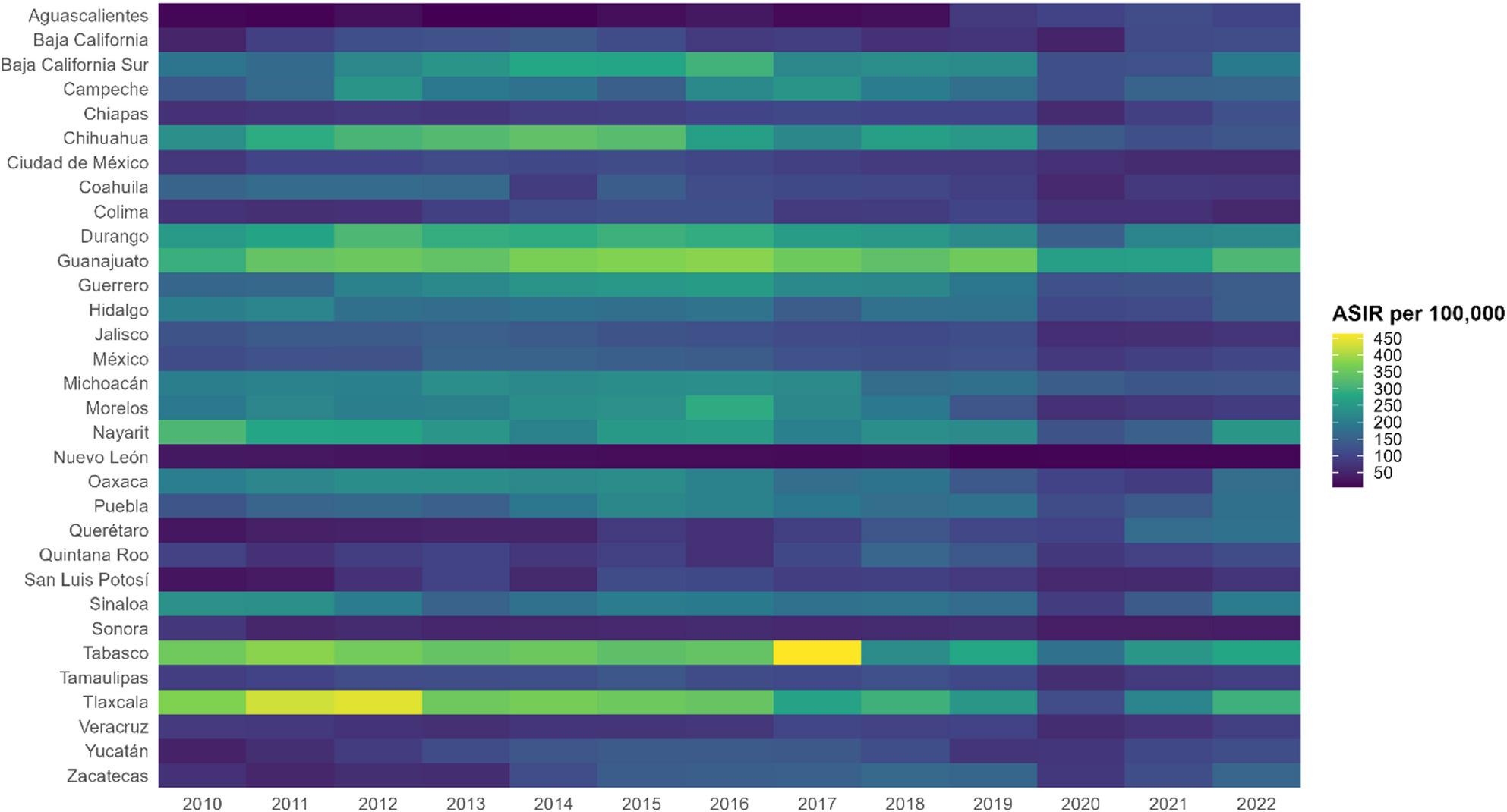
ASIR of surgical procedures per 100,000 children and adolescents stratified by state and year (n=752,654) from 2010-2022. The heat matrix displays age-standardized incidence rates (ASIR) of surgical procedures performed in Ministry of Health (MoH)-administered hospitals per 100,000 children and adolescents across Mexico’s 32 federal entities, stratified by year from 2010 to 2022. Darker tones (blue-purple) indicate lower procedure rates, while lighter tones (yellow) denote higher rates. Values range from 6.8 to 463.0 cases per 100,000 population aged under 18 years and reflect pediatric surgical utilization within the MoH public hospital system, rather than population-level surgical incidence across all healthcare sectors

The highest annual ASIR were recorded in Tabasco in 2017 (463.0), Tlaxcala in 2012 (441.9) and 2011 (429.2), and Guanajuato in 2016 (380.7), while the lowest annual rates occurred in Aguascalientes in 2013 (6.8), Nuevo León in 2019 (9.6) and Querétaro de Arteaga in 2010 (28.3) (Fig. 3 and Supplementary Table 5).

### Trend analysis of surgical procedures volume

**Fig. 4:**
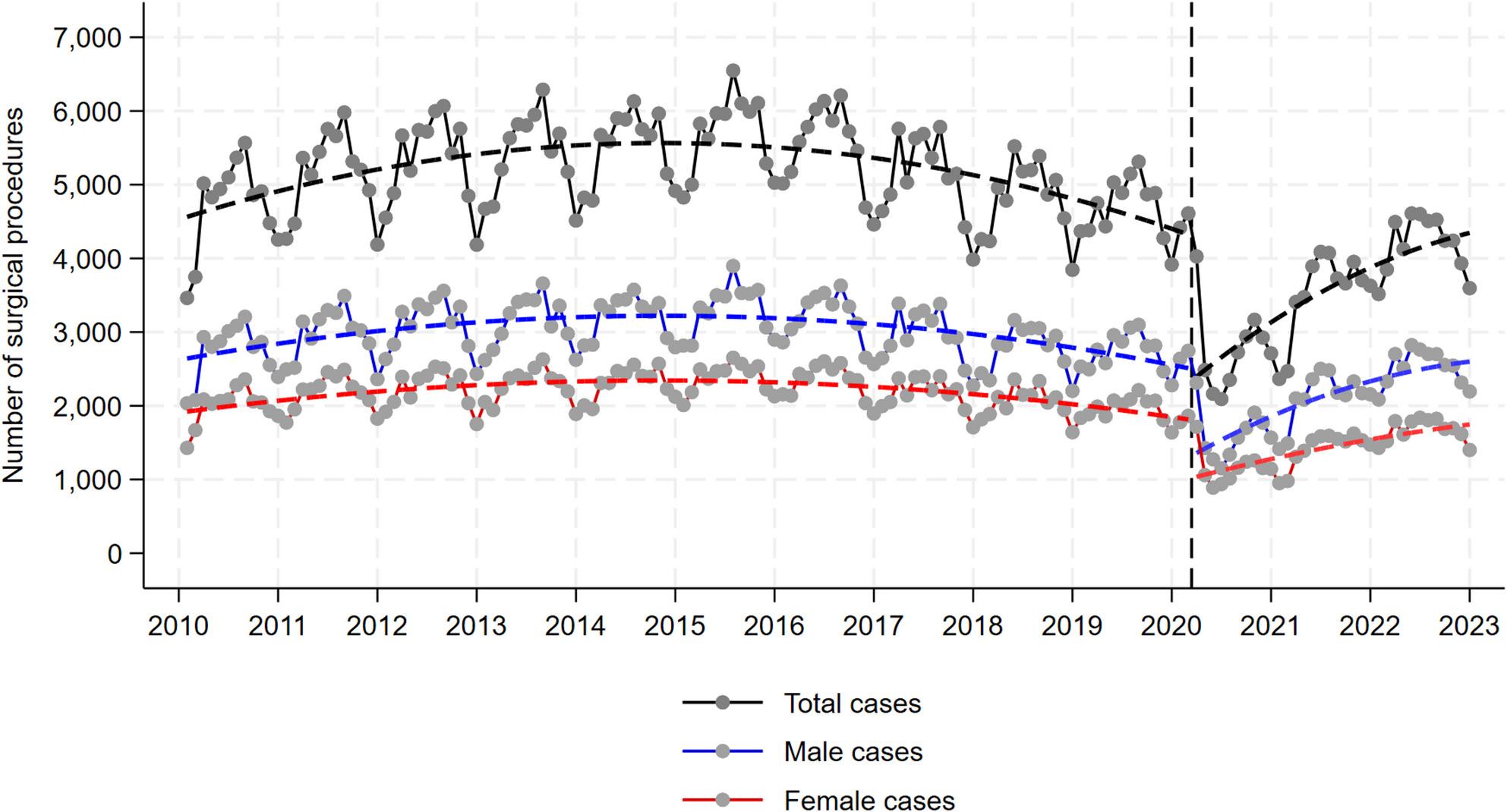
Trend analysis of surgical procedures in children and adolescents in Mexico from 2010-2022 (n=752,654). The dashed vertical line indicates the onset of the COVID-19 lockdown measures in Mexico (April 2020)

Surgical procedure volumes increased from a monthly average of 4,711.4 in 2010 to a peak of 5,689.4 in 2015 (+ 20.7%), followed by a decline to 4,688.7 in 2019 (− 17.6%), indicating a downward trend even before the onset of the COVID-19 pandemic **(**Fig. 4 and Supplementary Table 6) This pattern was consistent across sexes, with monthly volumes for male patients peaking at 3,307.2 in 2015 before declining to 2,727.8 in 2019 (− 17.5%), and for female patients peaking at 2,382.2 in 2015 before decreasing to 1,960.9 in 2019 (− 17.7%). ITS analysis showed that surgical procedure volumes declined sharply by 35.0% in April 2020 following the onset of the pandemic lockdowns in Mexico (exp(β): 0.65, 95% CI: 0.59–0.70) (Fig. 4 and Supplementary Table 7). During the pandemic period (April 2020-December 2022), surgical procedure volumes steadily increased to a monthly average of 4,186.5 in 2022, remaining below pre-pandemic levels. Sex-stratified ITS analysis showed a similar level drop in April 2020, with a 37.0% decline among male patients (exp(β): 0.63, 95% CI: 0.57–0.68) and a 35.0% decline among female patients (exp(β): 0.65, 95% CI: 0.60–0.70).

**Fig. 5:**
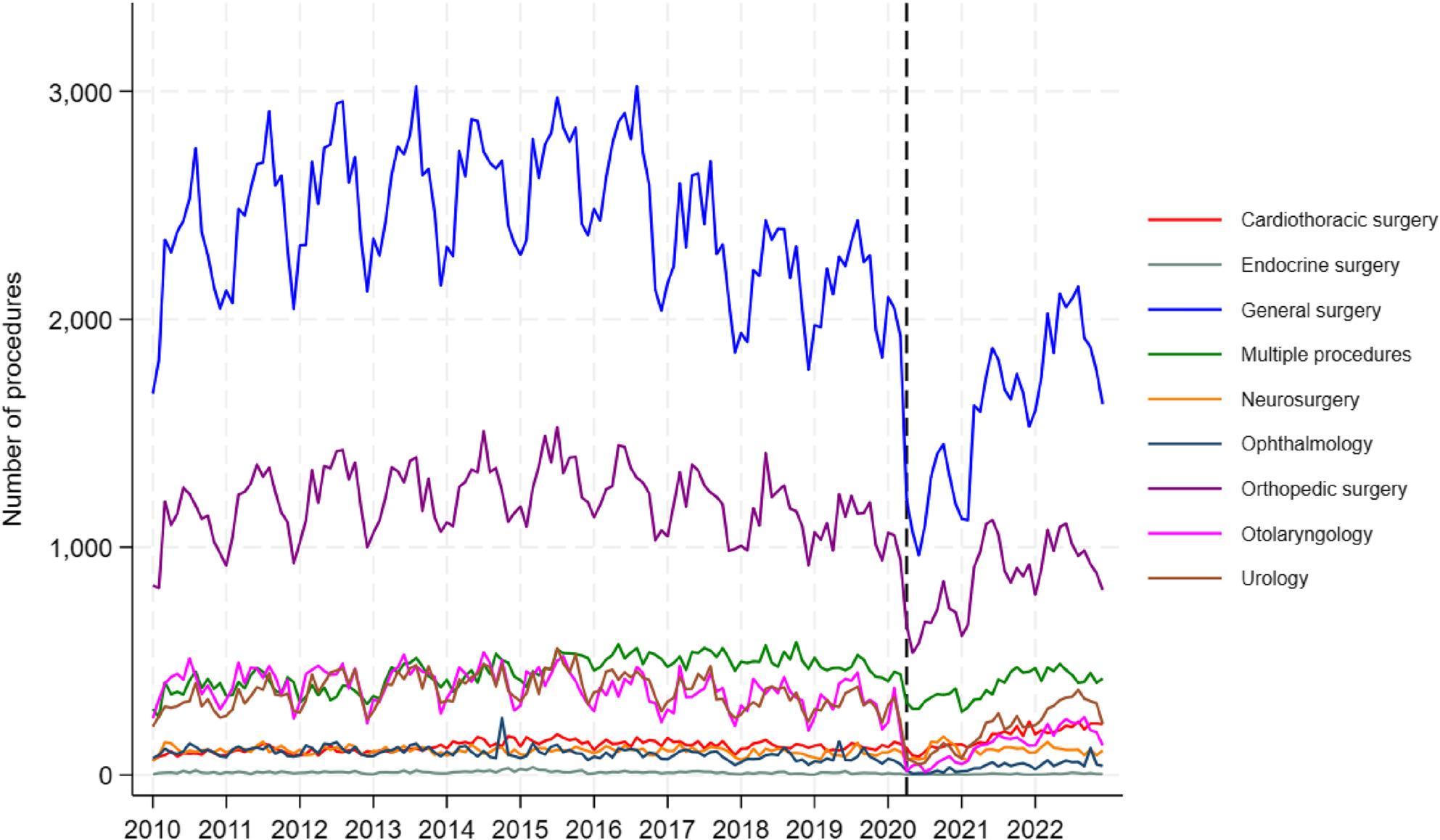
Trend analysis of surgical procedures from 2010-2022 in Mexico (n=752,654) stratified by surgical specialty. The dashed vertical line indicates the onset of the COVID-19 lockdown measures in Mexico (April 2020)

When analyzing trends for each surgical specialty separately, general surgery volumes dropped by 31.0% in April 2020 (exp(β): 0.69, 95% CI: 0.63–0.75) (Fig. 5 and Supplementary Table 8). In contrast, cardiothoracic surgery volumes did not show a significant level change in April 2020 (exp(β): 1.08, 95% CI:1.00-1.16) and average monthly cardiothoracic surgery volumes increased by 73.2% during the pandemic from 119.6 in 2020 to 207.1 in 2022 (Fig. 5 and Supplementary Table 9).

### Factors associated with in-hospital mortality

Children under 5 years exhibited significantly higher odds of in-hospital mortality compared to adolescents aged 15–17 years (aOR: 3.50, 95% CI: 3.18–3.86), as did underweight patients relative to those with normal weight (aOR: 2.05, 95% CI: 1.92–2.18) (Table 2). Indigenous identity was modestly associated with increased in-hospital mortality risk (aOR: 1.24, 95% CI: 1.03–1.50). ICU admission showed the strongest association with in-hospital mortality (aOR: 10.02, 95% CI: 7.87–12.74), followed by cardiothoracic surgery (aOR: 2.42, 95% CI: 2.21–2.65). Hospital-acquired infections (aOR: 2.71, 95% CI: 2.46–2.98) and prolonged length of stay (aOR: 2.27, 95% CI: 2.13–2.41) were also significant predictors.

**Table 2:** Predictors of in-hospital mortality among pediatric surgical patients in Mexico from 2010–2022 using multivariable logistic regression

	Univariable model		Multivariable model	
	OR	95% CI	aOR	95% CI
Sex				
Male	Reference category			
Female	1.05	1.00, 1.10	0.96	0.91, 1.02
Age category				
15–17 years	Reference category			
Under 5 years	7.50	6.94, 8.11	3.50	3.18, 3.86
5–9 years	0.56	0.49, 0.63	0.59	0.51, 0.68
10–14 years	0.72	0.65, 0.80	0.81	0.72, 0.91
BMI-for-age category				
Normal weight	Reference category			
Underweight	5.12	4.85, 5.41	2.05	1.92, 2.18
Overweight	0.80	0.74, 0.87	0.98	0.90, 1.08
Obese	0.83	0.77, 0.91	1.00	0.91, 1.09
Indigenous identity				
No	Reference category			
Yes	0.90	0.76, 1.07	1.24	1.03, 1.50
Ward type				
Surgery	Reference category			
ICU	50.26	41.21, 61.29	10.02	7.87, 12.74
Internal medicine	3.55	3.04, 4.14	3.54	2.94, 4.25
Pediatrics	1.37	1.30, 1.44	0.86	0.81, 0.92
Other	0.74	0.70, 0.79	1.04	0.97, 1.12
Missing	0.06	0.04, 0.09	-	-
Surgical procedure				
General surgery	Reference category			
Cardiothoracic Surgery	8.48	7.85, 9.15	2.42	2.21, 2.65
Endocrine Surgery	0.16	0.06, 0.44	0.14	0.05, 0.36
Multiple procedures	0.59	0.55, 0.63	0.42	0.39, 0.46
Neurosurgery	3.73	3.40, 4.09	0.97	0.88, 1.07
Ophthalmology	0.11	0.07, 0.17	0.07	0.05, 0.12
Orthopedic Surgery	0.04	0.03, 0.04	0.03	0.02, 0.04
Otolaryngology	0.04	0.03, 0.06	0.02	0.01, 0.03
Urology	0.21	0.18, 0.25	0.10	0.08, 0.12
Type of anesthesia				
General	Reference category			
Combined	0.34	0.29, 0.40	0.47	0.40, 0.55
Local	0.45	0.41, 0.49	0.48	0.43, 0.53
Regional	0.18	0.16, 0.19	0.32	0.30, 0.35
Sedation	0.93	0.85, 1.03	1.16	1.05, 1.29
Missing	2.85	2.66, 3.06	-	-
HAI				
No	Reference category			
Yes	13.11	12.11, 14.19	2.71	2.46, 2.98
LOS				
< 4 days	Reference category			
>= 4 days	6.50	6.17, 6.83	2.27	2.13, 2.41

*OR* Odds ratio, *95% CI* 95% Confidence interval, *aOR* Adjusted odds ratio

*BMI* Body mass index, *Ward* ward at admission; transfers during hospitalization are not captured, *ICU* Intensive care unit, *HAI* Hospital-acquired infection, *LOS* Length of stay

## Discussion

This study provides a comprehensive overview of pediatric surgical procedures performed in MoH-administered public hospitals across Mexico from 2010 to 2022. To our knowledge, it represents the first national analysis of pediatric surgical epidemiology in this setting. We describe over 750,000 procedures, reveal geographic inequities that demand attention, characterize temporal trends including the pre-pandemic decline and the impact of COVID-19, and identify factors associated with in-hospital mortality among children and adolescents. Our findings reveal substantial geographic disparities, with Guanajuato reporting the highest overall incidence (333.8 per 100,000) and Nuevo León the lowest (18.5 per 100,000), illustrating persistent inequities in access to surgical care for children. We observed an increase in surgical procedure volumes from 2010, peaking in 2015, followed by a decline and an abrupt 35% drop during the COVID-19 pandemic, with gradual recovery by 2022. Factors associated with increased in-hospital mortality included age under 5 years, underweight, ICU admission, cardiothoracic surgery, HAIs, and prolonged LOS. Additionally, overweight and obesity were common, affecting 41% of pediatric surgical patients. These findings underscore the need for targeted, evidence-informed interventions to address the most affected regions and vulnerable patient groups, thereby reducing pediatric health disparities in access to surgical care. Across MoH-administered hospitals, these findings point to feasible, system-level interventions, including strengthening referral pathways, improving perioperative and infection prevention practices, and enhancing routine data use for quality monitoring, as pragmatic strategies to improve pediatric surgical outcomes within existing resource constraints.

### Geographic variations in surgical procedure volumes

The highest surgical utilization rate was observed in the Mexican state of Guanajuato (333.8 per 100,000), while the lowest was found in Nuevo León (18.5 per 100,000). Because our analysis is restricted to Ministry of Health hospitals, these disparities primarily reflect the experience of children and adolescents whose families lack employment-based social security and rely on the MoH system. Accordingly, the reported ASIRs should be interpreted as indicators of pediatric surgical utilization within the MoH subsystem rather than true population-level surgical incidence. For example, the contrast observed between Guanajuato, which showed the highest ASIR, and Nuevo León, which showed the lowest ASIR in this analysis, may partly reflect differences in the distribution of healthcare subsystems, where pediatric surgical care in some states is more frequently delivered through IMSS or private hospitals rather than MoH-administered facilities, rather than reflecting differences in underlying surgical need. These findings are consistent with Pérez-Soto et al., who reported similar patterns across all age groups in Mexico in 2020 [30]. Guanajuato’s highly organized healthcare system ensures that 99.8% of the population has access to essential services, which likely facilitates a higher surgical volume [56]. Supporting this, Guanajuato also reports more surgical specialists per 100,000 inhabitants (124.0) than Nuevo León (110.7), and a greater proportion of the population with access to surgical care within two hours (87.8% vs. 81.1%) [30]. These geographic disparities highlight persistent inequities in pediatric surgical access across Mexico.

Differing documentation practices may further contribute to these variations. Guanajuato’s Institute of Planning, Statistics, and Geography (Instituto de Planeación, Estadística y Geografía, INEGI) supplements the automated hospital discharge database [SAEH] with geographic information system (GIS) mapping and structured user surveys to verify service availability and quality [57]. Expanding such integrated approaches to underperforming regions could enhance registry completeness and guide resource allocation [57]. The comparatively higher surgical utilization rates rate among children than adolescents in Mexico City reflects the centralization of specialized pediatric services, whereby infants and young children with congenital anomalies and other complex conditions are referred to tertiary centers in the capital for surgical treatment [58, 59].

### Temporal trends of surgical procedure volumes

Surgical procedure volumes gradually increased from 2010, peaking in 2015, before declining even prior to the COVID-19 pandemic. This pre-pandemic decrease temporally coincided with the later years of the Seguro Popular program, a period characterized by stagnating budgets and administrative challenges, including reported funding shortfalls and procurement disruptions across public hospitals [60, 61]. However, our analysis does not directly evaluate health financing or policy reform variables, and therefore these observations should be interpreted as contextual associations rather than causal effects.

In April 2020, at the onset of lockdown measures in Mexico, surgical volume dropped by 35.0%, reflecting a combination of reduced surgical capacity and decreased demand [31, 62]. In contrast, procedure volumes for some acute and high-complexity surgical specialties like cardiothoracic surgery did not show a significant decline in April 2020, reflecting the prioritization of life-saving interventions during the pandemic [63]. These specialty-specific patterns provide insight into system-level prioritization decisions during a national health system shock, rather than merely reflecting overall cancellation of elective procedures. Many public hospitals in Mexico repurposed operating spaces for COVID-19 care, further limiting surgical infrastructure [64], and tertiary centers reported drastic reductions in surgical procedures [65]. In parallel, studies from Colombia, Italy, and Canada documented sharp declines in pediatric emergency visits and trauma admissions, suggesting both fewer surgical indications and widespread hospital avoidance due to infection fears [66–68]. Our findings are consistent with other studies from Mexico and LMICs that reported significant reductions in pediatric surgical volume during the pandemic [33, 69]. One multicenter study across four LMICs (Burkina Faso, Ecuador, Nigeria, and Zambia) found a 32% decline in pediatric surgical cases from 2019 to 2020 [33], while a Mexican hospital reported a 79.3% decrease in total surgical activity (children and adults) [69].

Mexico’s response combined extended operating hours and public-private partnerships to address surgical backlog. Mexico’s public sector, later including IMSS-Bienestar, established public-private partnerships and implemented night and weekend shifts, while some states, such as Baja California, outsourced high-volume, low-complexity procedures to private providers in early 2021 [70, 71]. These measures mirror broader Latin American trends: a scoping review found that 41% of institutions adopted prioritization protocols to safely resume elective procedures [72]. Colombia implemented a phased reopening with strict infection control and epidemiologic monitoring [73], which resulted in economic and healthcare services being gradually restored in stages based on local transmission rates and healthcare capacity. Brazil launched data-driven recovery plans bolstered by supplemental funding [74].

### Mortality and associated risk factors

In our study, the overall in-hospital mortality rate was 1.0%. This rate is relatively low compared with mortality reported in many pediatric surgical cohorts from low- and middle-income countries, where perioperative or in-hospital mortality rates frequently range from approximately 3% to over 10% depending on case mix and resource availability [75–77].

The increased in-hospital mortality associated with cardiothoracic surgery in our analysis is consistent with previous research showing that complex pediatric procedures carry substantially higher risk [78, 79]. Data from Mexico’s national pediatric cardiac surgery registry reported a 7.5% mortality rate among children undergoing cardiothoracic operations [80]. Even higher rates have been documented in other low- and middle-income countries (LMICs), including Indonesia (13.6%) and China (16.3%) [78, 79], while a large multicenter study from the United States reported a lower overall rate of 3.2% [81]. The elevated mortality observed in children under 5 years of age in our study may, in part, reflect the disproportionate burden of cardiothoracic procedures performed in this age group, particularly in neonates, who face inherently greater surgical risk [82]. In Mexico, national strategies to improve outcomes have included regionalizing pediatric cardiac surgery to high-capacity centers and standardizing care protocols [83]. Although ICU admissions were rare (0.1%), ICU admission was strongly associated with in-hospital mortality, likely reflecting severe case selection and potential underreporting of ICU utilization in administrative hospital discharge data, and may partly reflect the concentration of high-risk procedures such as cardiothoracic surgery within critically ill patients. Because ICU status in this analysis reflects the hospital service recorded at admission, patients who required ICU care later during hospitalization may still be recorded under another ward type, which may contribute to the low proportion of ICU admissions observed. Indigenous identity was also associated with increased in-hospital mortality. This finding should be interpreted within a broader equity framework. Indigenous populations in Mexico experience persistent structural barriers to healthcare, including geographic isolation, residence in highly marginalized municipalities, limited availability of specialized services, and longer travel distances to hospitals [84, 85]. These barriers may contribute to delayed presentation, more advanced disease at admission, and reduced access to timely surgical and perioperative care [86, 87]. Linguistic, cultural, and discriminatory barriers within public services may further discourage early care-seeking and continuity of care [88, 89]. Together, these factors suggest that the observed excess in-hospital mortality reflects systemic inequities rather than intrinsic patient-level risk. These findings underscore the need for equity-focused strategies that address access, referral pathways, and culturally safe care for indigenous children. At the same time, indigenous identity may be underreported in administrative hospital data and likely functions as a proxy for broader structural determinants such as geographic marginalization and delayed access to care [89–91]. Therefore, the observed association should be interpreted with consideration of potential misclassification and residual confounding.

In our study, HAIs were also strongly associated with increased in-hospital mortality among pediatric surgical patients in Mexico. This finding aligns with other evidence from Mexico, including a study reporting that pediatric surgery patients with nosocomial infections had approximately four times the risk of death compared to their uninfected counterparts [92]. Similarly, a recent Mexican study of pediatric burn injury patients found that children and adolescents with in-hospital infections had nearly sixfold higher odds of mortality [93]. These results highlight the urgent need for improved infection control in pediatric surgical care. Implementing multimodal strategies, including strict hand hygiene, catheter care protocols, and adherence to WHO’s *Clean Care is Safer Care* campaign, which promotes global patient safety through improved infection prevention practices, has been shown to significantly reduce HAIs and related mortality in hospital settings [94]. Within MoH-administered hospitals, these findings support the feasibility of strengthening low-cost, evidence-based infection prevention interventions, such as standardized perioperative protocols, routine HAI surveillance, and targeted staff training, to reduce preventable pediatric surgical mortality.

Moreover, the association between prolonged length of stay and in-hospital mortality should be interpreted as reflecting greater case severity, postoperative complications, or the need for extended monitoring and care, rather than as an independent risk factor for mortality.

Finally, the finding that the “multiple procedures” category was associated with lower in-hospital mortality is counterintuitive and should not be interpreted as a protective effect of undergoing more than one procedure. Because this category captures any admission with more than one ICD-9-CM procedure code without distinguishing timing, urgency, or procedural complexity, it often aggregates heterogeneous procedure types.

### Obesity and overweight among pediatric surgical patients

In our cohort, 41.0% of children and adolescents were overweight or obese, which aligns with national estimates from the year 2022 from Mexico’s nationally representative health and nutrition survey *(Encuesta Nacional de Salud y Nutrición*, ENSANUT) [95] and other national Mexican studies on childhood obesity [96, 97]. While Mexico has implemented important policy measures such as a sugar-sweetened beverage tax and front-of-package warning labels, further progress is needed to effectively reduce pediatric obesity [98]. In Chile, a comprehensive policy approach combining mandatory restrictions on child-directed marketing and bans on the sale of unhealthy foods in schools led to a 73% reduction in children’s exposure to junk food advertising and a measurable decline in purchases of sugar-sweetened products [99]. Given that pediatric obesity is consistently associated with worse surgical outcomes and increased perioperative complications, including infections, prolonged operating time and anesthetic risk [100–102], policy responses should also address obesity prevention and management overall and within surgical care planning.

### Limitations and strengths

This study has several limitations. The data are limited to MoH-administered hospitals, which account for approximately 30% of hospitals nationwide and primarily serve children and adolescents without private insurance or employment-based social security. While findings are not generalizable to all pediatric surgical care in Mexico, they are highly relevant to the public health system responsible for delivering surgical services to socioeconomically vulnerable populations. As a result, our findings primarily reflect the experience of children and adolescents whose families lack employment-based social security and depend on the MoH system and may not be fully generalizable to the broader pediatric population in Mexico. For example, the contrast observed between Guanajuato and Nuevo León may partly reflect differences in where pediatric surgical care is delivered across health subsystems, rather than differences in underlying surgical need.

Additionally, the use of administrative registry data may introduce biases related to coding accuracy and missing or incomplete records, and the absence of detailed clinical information precludes monitoring of patient outcomes or quality of care over time. “Furthermore, the registry does not capture facility-level indicators of pediatric surgical infrastructure or workforce capacity. Consequently, this analysis cannot assess facility-level pediatric surgical capacity or evaluate gaps in specialized pediatric surgical services within the MoH hospital system. The registry does not contain sufficient detail to stratify procedures according to operative technique or procedural complexity. Consequently, procedures were analyzed within broader specialty-based categories, and differences in procedural complexity could not be evaluated in this analysis. Excluding readmissions underestimates procedure volumes and complications that occur during subsequent encounters; however, it reduces intra-patient correlation and double counting. In addition, ICU status in this analysis reflects the hospital service recorded at admission, and therefore patients transferred to ICU later during hospitalization are not fully captured.

Because the dataset does not include variables capturing health system financing or policy implementation, it was not possible to disentangle the independent effects of health system reforms (including the transition from Seguro Popular to INSABI) from broader system disruptions associated with the COVID-19 pandemic when interpreting temporal changes in surgical volume. Furthermore, the registry does not contain information on the specialty of the operating surgeon or on referral pathways. Therefore, this analysis cannot determine whether procedures were performed by pediatric surgeons or general surgeons, nor can it assess referral patterns or patient transfers between facilities, as the dataset does not allow tracking patients across different levels of care. This is an important consideration given the uneven distribution of the surgical workforce and access to surgical care across Mexico [30].

Several limitations related to missing data should also be noted. Records with missing demographic information, including indigenous identity, and with missing or implausible anthropometric data, were excluded. We descriptively analyzed exclusions related to missing or implausible BMI-for-age values; however, these exclusions may have influenced estimates of nutritional status and associated outcomes if BMI missingness was not random. Underreporting or incomplete documentation of indigenous identity may lead to underestimation of inequities in surgical access and outcomes among populations that face well-documented health inequities in Mexico [84, 103]. These exclusions may have shifted the composition of the analysis population toward hospitals and patient groups with more complete documentation, potentially limiting representativeness. Moreover, differential patterns of missingness across states or hospital types could influence observed geographic and clinical differences.

Despite these limitations, this study provides the first national analysis of pediatric surgical care in Mexico, using over a decade of data to characterize geographic and temporal patterns in surgical delivery and identify disparities that need urgent attention. Its findings offer a valuable foundation for planning, policy, and future research on surgical access and equity.

## Conclusion

This national analysis highlights significant geographic and temporal inequalities and inequities in pediatric surgical care across Mexico’s public hospitals from 2010 to 2022. Our findings underscore the need for context-specific strategies to strengthen surgical care for children. Analysis of temporal trends reveals a pre-pandemic decline in pediatric surgical volume, followed by a sharp disruption during the COVID-19 pandemic and an incomplete recovery by 2022. In addition, we identify key factors associated with in-hospital mortality, including younger age, cardiothoracic surgery, ICU admission, hospital-acquired infections, and prolonged length of stay, underscoring vulnerable patient groups and modifiable system-level risks.

Together, these findings emphasize the need for context-specific strategies to strengthen pediatric surgical care in Mexico, including investments in workforce development, equitable referral pathways, infection prevention, and data systems capable of supporting evidence-based policy. By providing the first national analysis of pediatric surgical care in this setting, this study offers critical insights to guide targeted policy and investment decisions aimed at improving equity, resilience, and outcomes for children across Mexico. Future research should incorporate patient-level outcomes, surgical quality indicators, and access barriers to support ongoing monitoring and system improvement. 

## Supplementary Information

Supplementary Material 1.

## Data Availability

The hospital discharge database used in this paper was generated from Mexico’s Automated Hospital Discharge System (Subsistema Automatizado de Egresos Hospitalarios, SAEH) and is publicly available through the Mexican Health Ministry at: [http://www.dgis.salud.gob.mx/contenidos/sinais/s_saeh.html](http://www.dgis.salud.gob.mx/contenidos/sinais/s_saeh.html) .The dataset used in this study was downloaded on February 22, 2025. The website is currently not accessible, but the data were fully available at the time of download.

## Abbreviations

BMI: Body mass index
CCS: Clinical Classifications Software
COVID-19: Coronavirus disease 2019
HAI: Hospital-acquired infection
ICD-9: International Classification of Diseases, 9th Revision
IMSS: Instituto Mexicano del Seguro Social (Mexican Social Security Institute)
IMSS-Bienestar: Instituto Mexicano del Seguro Social-Bienestar (Mexican Social Security Institute for Well-being)
INSABI: Instituto de Salud para el Bienestar (Institute of Health for Well-being)
IRB: Institutional Review Board
IQR: Interquartile range
LOS: Length of stay
MoH: Ministry of Health
SAEH: Subsistema Automatizado de Egresos Hospitalarios (Automated Hospital Discharge System)
SARS-CoV-2: Severe acute respiratory syndrome coronavirus 2
SSA: Secretaría de Salud (Secretariat of Health)
WHO: World Health Organization

## References

[1] Bickler SN, Weiser TG, Kassebaum N, Higashi H, Chang DC, Barendregt JJ, et al. Global Burden of Surgical Conditions. In: Debas HT, Donkor P, Gawande A, Jamison DT, Kruk ME, Mock CN, editors. Essential Surgery: Disease Control Priorities, Third Edition. Volume 1. Washington (DC): The International Bank for Reconstruction and Development / The World Bank; 2015. pp. 19–40. 26740991

[2] Ozgediz D, Poenaru D. The burden of pediatric surgical conditions in low and middle income countries: a call to action. J Pediatr Surg. 2012;47:2305–11. 10.1016/j.jpedsurg.2012.09.030. 23217895 10.1016/j.jpedsurg.2012.09.030

[3] Meara JG, Leather AJM, Hagander L, Alkire BC, Alonso N, Ameh EA, et al. Global Surgery 2030: evidence and solutions for achieving health, welfare, and economic development. Lancet. 2015;386:569–624. 10.1016/j.ijoa.2015.09.006. 25924834 10.1016/S0140-6736(15)60160-X

[4] Mullapudi B, Grabski D, Ameh E, Ozgediz D, Thangarajah H, Kling K, et al. Estimates of number of children and adolescents without access to surgical care. Bull World Health Organ. 2019;97:254–8. 10.2471/BLT.18.216028. 30940982 10.2471/BLT.18.216028 PMC6438256

[5] Pulvirenti R, Gortan M, Cumba D, Gamba P, Tognon C. Pediatric Surgery and Anesthesia in Low-Middle Income Countries: Current Situation and Ethical Challenges. Front Pediatr. 2022;10. 10.3389/fped.2022.908699. 10.3389/fped.2022.908699 PMC9369455 35967563

[6] Wasserman I, Peters AW, Roa L, Amanullah F, Samad L. Breaking Specialty Silos: Improving Global Child Health Through Essential Surgical Care. Glob Health Sci Pract. 2020;8:183–9. 10.9745/GHSP-D-20-00009. 32606090 10.9745/GHSP-D-20-00009 PMC7326524

[7] Alkire BC, Raykar NP, Shrime MG, Weiser TG, Bickler SW, Rose JA, et al. Global access to surgical care: a modelling study. Lancet Glob Health. 2015;3:e316–23. 10.1016/S2214-109X(15)70115-4. 25926087 10.1016/S2214-109X(15)70115-4 PMC4820251

[8] Greenberg SLM, Cockrell HC, Hyman G, Goodman L, Kaseje N, Oldham KT. The Global Initiative for Children’s Surgery: conception, gestation, and delivery. Pediatr Surg Int. 2022;39:48. 10.1007/s00383-022-05319-4. 36507955 10.1007/s00383-022-05319-4 PMC9744037

[9] United Nations, Department of Economic and Social Affairs. THE 17 GOALS | Sustainable Development. 2015. https://sdgs.un.org/goals. Accessed 25 Sept 2025.

[10] Block M, Morales HR, Balandrán A, Méndez E. Mexico: health system review. 2020. https://iris.who.int/handle/10665/334334. Accessed 13 Sept 2024.

[11] Homedes N, Ugalde A. Twenty-Five Years of Convoluted Health Reforms in Mexico. PLoS Med. 2009;6:100–24. 10.1371/journal.pmed.1000124. 10.1371/journal.pmed.1000124 PMC2719806 19688039

[12] Gómez-Dantés O, Flamand L, Cerecero-García D, Morales-Vazquez M, Serván-Mori E. Origin, impacts, and potential solutions to the fragmentation of the Mexican health system: a consultation with key actors. Health Res Policy Syst. 2023;21:80. 10.1186/s12961-023-01025-2. 37525130 10.1186/s12961-023-01025-2 PMC10388521

[13] Gómez-Dantés O, Serván-Mori E, Cerecero D, Flamand L, Mohar A. Mexico’s Health System, 2023. Salud Publica Mex. 2024;67:91–105. 10.21149/15802. 39977189 10.21149/15802

[14] Coneval C. Comunicado de prensa No. 07, El Coneval presenta las estimaciones de pobreza multidimensional 2022. 2023. https://www.coneval.org.mx/SalaPrensa/Comunicadosprensa/Documents/2023/Comunicado_07_Medicion_Pobreza_2022.pdf#:~:text=%E2%80%A2%20El%20porcentaje%20de%20personas,7. Accessed 28 June 2025.

[15] Puentes-Rosas E, Sesma S, Gómez-Dantés O. Estimated Population with Health Insurance in Mexico Based on a National Survey. Salud Publica Mex. 2005;47:22–6. 16101203

[16] Gómez-Dantés O, Sesma S, Becerril VM. Sistema de salud de México. Salud Publica Mex. 2011;53:220–32. 21877087

[17] Frenk J, Gómez-Dantés O. Health System in Mexico. Health Services Evaluation. New York, NY: Springer; 2019. pp. 849–59. 10.1007/978-1-4939-8715-3_46.

[18] Knaul FM, Arreola-Ornelas H, Touchton M, McDonald T, Blofield M, Burgos LA, et al. Setbacks in the quest for universal health coverage in Mexico: polarised politics, policy upheaval, and pandemic disruption. Lancet. 2023;402:731–46. 10.1016/S0140-6736(23)00777-8. 37562419 10.1016/S0140-6736(23)00777-8

[19] Rodriguez-Romo L, Olaya Vargas A, Gupta S, Shalkow-Klincovstein J, Vega-Vega L, Reyes-Lopez A, et al. Delivery of Pediatric Cancer Care in Mexico: A National Survey. J Glob Oncol. 2018;4:1–12. 10.1200/JGO.17.00238. 30084750 10.1200/JGO.17.00238 PMC6223522

[20] Andrade R. IMSS-Bienestar is officially constituted. 2022. https://mexicobusiness.news/health/news/imss-bienestar-officially-constituted. Accessed 15 June 2025.

[21] Flamand L, Moreno-Jaimes C. La protección social en salud durante el gobierno de Calderón. Avances y rezagos en el diseño y la implementación del Seguro Popular (2006–2012). Foro Int. 2015;55:217–61.

[22] Secretaría de Salud. Acuerdo por el que la Secretaría de Salud da a conocer las Reglas de operación e indicadores de gestión y evaluación del Programa. 2007. https://www.coahuilatransparente.gob.mx/acuerdos/documentos_acuerdos/ISSREEIECAcuerdos121.pdf. Accessed 17 May 2025.

[23] Instituto Mexicano del Seguro Social. Diario Oficial de la Federación. 2023. https://www.dof.gob.mx/nota_detalle.php?codigo=5713366&fecha=29/12/2023#gsc.tab=0. Accessed 28 June 2025.

[24] Baeza-Herrera C, Velázquez-Pino H, Alarcón-Quezada V, Cortés-García R, García-Cabello LM. Satisfacción con la residencia de Cirugía Pediátrica en México: Resultados de una encuesta de opinión. Acta pediátrica de México. 2014;35:441–7.

[25] Magaña GM, Elton TMA, Moreno AA, Faúndez MP, Black EV, Rivera BM. The reality of neonatal surgery in Latin America and the Caribbean: Perspectives and opinions of pediatric surgeons. J Pediatr Surg Open. 2025;12:100234. 10.1016/j.yjpso.2025.100234.

[26] UNICEF. How many children are there in Mexico? UNICEF DATA. 2022. https://data.unicef.org/how-many/how-many-children-under-18-are-there-in-mexico/. Accessed 2 July 2023.

[27] Sandoval NF. Evaluación de la calidad en cirugía cardiovascular pediátrica. Rev Colomb Cardiol. 2012;19:277–80.

[28] Fernández-Ortega G, Morón-García GC, García-Sosa LE, Suárez-Delgadillo MS, Hinojosa-Velasco A. Ten years of pediatric surgery in a secondary level perinatal hospital in Mexico. Bol Med Hosp Infant Mex. 2023;80:302–11. 37963301 10.24875/BMHIM.22000159

[29] González G, Pacheco-Cesar H, González-Woge M, Shalkow-Klincovstein J, Lobos PA, Valle PL-D, et al. Pediatric surgery in Latin America: Current realities, challenges, and future directions. J Pediatr Surg Open. 2026;13:100243. 10.1016/j.yjpso.2025.100243.

[30] Pérez-Soto RH, Trolle-Silva AM, Buerba-Romero Valdés GA, Sánchez-Morales GE, Velázquez-Fernández D, Ramos-De la Medina A, et al. Timely Access to Essential Surgery, Surgical Workforce, and Surgical Volume: Global Surgery Indicators in Mexico. Glob Health Sci Pract. 2023;11:e2100745. 10.9745/GHSP-D-21-00745. 36853648 10.9745/GHSP-D-21-00745 PMC9972376

[31] COVIDSurg C. Elective surgery cancellations due to the COVID-19 pandemic: global predictive modelling to inform surgical recovery plans. Br J Surg. 2020;107:1440–9. 10.1002/bjs.11746. 32395848 10.1002/bjs.11746 PMC7272903

[32] Søreide K, Hallet J, Matthews JB, Schnitzbauer AA, Line PD, Lai PBS, et al. Immediate and long-term impact of the COVID-19 pandemic on delivery of surgical services. Br J Surg. 2020;107:1250–61. 10.1002/bjs.11670. 32350857 10.1002/bjs.11670 PMC7267363

[33] Park P, Laverde R, Klazura G, Yap A, Bvulani B, Ki B, et al. Impact of the COVID-19 Pandemic on Pediatric Surgical Volume in Four Low- and Middle-Income Country Hospitals: Insights from an Interrupted Time Series Analysis. World J Surg. 2022;46:984–93. 10.1007/s00268-022-06503-2. 35267077 10.1007/s00268-022-06503-2 PMC8908743

[34] Secretaría de Salud. Datos Abiertos de México - Egresos Hospitalarios de la Secretaría de Salud. https://datos.gob.mx/busca/dataset/egresos-hospitalarios-de-la-secretaria-de-salud. Accessed 8 Mar 2025.

[35] Instituto Nacional de Estadística y Geografía. Censo de Población y Vivienda 2020: CPV: principales resultados : Estados Unidos Mexicanos / Instituto Nacional de Estadística y Geografía.-- México. Census. Mexico; 2021.

[36] Holland & Knight. Mexico Declares COVID-19 Sanitary Emergency by Reason of Force Majeure. https://www.hklaw.com/en/insights/publications/2020/04/mexico-declares-covid19-sanitary-emergency-by-reason-of-force-majeure. Accessed 6 June 2025.

[37] World Health Organization. WHO child growth standards: length/height-for-age, weight-for-age, weight-for-length, weight-for-height and body mass index-for-age: methods and development. 2006. https://www.who.int/publications/i/item/924154693X. Accessed 27 May 2025.

[38] World Health Organization. Growth reference 5–19 years - BMI-for-age (5–19 years). 2006. https://www.who.int/tools/growth-reference-data-for-5to19-years/indicators/bmi-for-age. Accessed 28 May 2025.

[39] Martínez C, Telles E, Torche F. From language to self-identification: indigenous classification in the Americas. Soc Forces. 2025;soaf152. 10.1093/sf/soaf152.

[40] World Health Organization. International Classification of Diseases: 9th Revision. Geneva: World Health Organization; 1978.

[41] Agency for Healthcare Research and Quality (AHRQ). Clinical Classification - Procedure Categories. 2024. https://hcup-us.ahrq.gov/toolssoftware/ccs/AppendixDMultiPR.txt. Accessed 12 Feb 2025.

[42] Monegro AF, Muppidi V, Regunath H. Hospital-Acquired Infections. StatPearls. Treasure Island (FL). StatPearls Publishing; 2025. 28722887

[43] Butler LR, Dominy CL, White CA, Mengsteab P, Lin E, Allen AK, et al. Risk factors for 90-day readmission and prolonged length of stay after hip surgery in children with cerebral palsy. J Orthop. 2023;38:14–9. 10.1016/j.jor.2023.03.002. 36925762 10.1016/j.jor.2023.03.002 PMC10011680

[44] Wang Y, Honeyford K, Aylin P, Bottle A, Giuliani S. One-year outcomes for congenital diaphragmatic hernia. BJS Open. 2019;3:305–13. 10.1002/bjs5.50135. 31183446 10.1002/bjs5.50135 PMC6551417

[45] Gross TS, McCracken C, Heiss KF, Wulkan ML, Raval MV. The contribution of practice variation to length of stay for children with perforated appendicitis. J Pediatr Surg. 2016;51:1292–7. 10.1016/j.jpedsurg.2016.01.016. 26891834 10.1016/j.jpedsurg.2016.01.016

[46] Eid SM, Ponor L, Reed DA, Beydoun MA, Beydoun HA, Wright S. Associations Between In-Hospital Mortality, Health Care Utilization, and Inpatient Costs With the 2011 Resident Duty Hour Revision. J Grad Med Educ. 2019;11:146–55. 10.4300/JGME-D-18-00415.1. 31024645 10.4300/JGME-D-18-00415.1 PMC6476098

[47] Crespi CM, Gao S, Payne A, Nobari TZ, Avila A, Nau C, et al. Longitudinal trajectories of adiposity-related measures from age 2–5 years in a population of low-income Hispanic children. Pediatr Res. 2021;89:1557–64. 10.1038/s41390-020-1099-8. 32750702 10.1038/s41390-020-1099-8 PMC8163600

[48] Lewis KH, Skelton J, Hsu F-C, Ezouah P, Taveras EM, Block JP. Use of Electronic Health Record Data to Study the Association of Sugary Drink Consumption With Child Weight Status. Acad Pediatr. 2020;20:767–75. 10.1016/j.acap.2019.11.002. 31712182 10.1016/j.acap.2019.11.002

[49] Freedman DS, Lawman HG, Skinner AC, McGuire LC, Allison DB, Ogden CL. Validity of the WHO cutoffs for biologically implausible values of weight, height, and BMI in children and adolescents in NHANES from 1999 through 2012. Am J Clin Nutr. 2015;102:1000–6. 10.3945/ajcn.115.115576. 26377160 10.3945/ajcn.115.115576 PMC4631693

[50] Ahmad OB, Boschi-Pinto C, Lopez AD, Murray CJ, Lozano R, Inoue M. Age Standardization of Rates: A new WHO Standard. Geneva: World Health Organization; 2001.

[51] Vaughn Kelso N. Natural Earth - Free vector and raster map data. 2021. https://www.naturalearthdata.com/. Accessed 21 Sept 2025.

[52] Doubova SV, Leslie HH, Kruk ME, Pérez-Cuevas R, Arsenault C. Disruption in essential health services in Mexico during COVID-19: an interrupted time series analysis of health information system data. BMJ Glob Health. 2021;6:e006204. 10.1136/bmjgh-2021-006204. 34470746 10.1136/bmjgh-2021-006204 PMC8413469

[53] Borges G, García JA, Monroy-Nasr Z, Suicide. Covid-19 in Mexico: an update to 2021. Salud Publica Mex. 2023;65:402–6. 10.21149/14696. 38060894 10.21149/14696

[54] Schauer JM, Lee J, Diaz K, Pigott TD. On the bias of complete- and shifting-case meta-regressions with missing covariates. Res Synthesis Methods. 2022;13:489–507. 10.1002/jrsm.1558. 10.1002/jrsm.1558 PMC9545321 35343067

[55] Ashner MC, Garcia TP. Understanding the implications of a complete case analysis for regression models with a right-censored covariate. Am Stat. 2024;78:335–44. 10.1080/00031305.2023.2282629. 39070115 10.1080/00031305.2023.2282629 PMC11281394

[56] World Bank. Mexico State-Level Public Expenditure Review: The Case of Guanajuato. 2020. https://documents1.worldbank.org/curated/pt/369571468281722501/pdf/Mexico-State-level-public-expenditure-review-the-case-of-Guanajuato.pdf. Accessed 8 Mar 2025.

[57] Institute of Planning, Statistics, and Geography of the State of Guanajuato. IPLANEG. 2022. https://iplaneg.guanajuato.gob.mx/. Accessed 17 June 2025.

[58] Calderón-Colmenero J, De-la-Llata M, Vizcaíno A, Ramírez S, Bolio A. Medical and surgical health care for congenital heart disease: a panoramic vision of the reality in Mexico. Rev Invest Clin. 2011;63:344–52. 22364033

[59] Pizarro-Altamirano MF, Trousselle-Peralta M, Soriano-Rosales RE, Carmona-Librado S, Esparza-Aguilar M, Villagomez-Martinez SM et al. Epidemiological changes in child surgical activity. Analysis of 17 years in a tertiary care hospital in Mexico. 2020. https://www.researchsquare.com/article/rs-12415/v1. Accessed 29 Aug 2024.

[60] Serván-Mori E, Gómez-Dantés O, Contreras D, Flamand L, Cerecero-García D, Arreola-Ornelas H, et al. Increase of catastrophic and impoverishing health expenditures in Mexico associated to policy changes and the COVID-19 pandemic. J Glob Health. 2023;13:06044. 10.7189/jogh.13.06044. 37883200 10.7189/jogh.13.06044 PMC10602209

[61] McDonald T, Touchton M, Knaul FM, Arreola-Ornelas H, Frenk J. The Rise and Fall of Seguro Popular: Mexico’s Health Care Odyssey. Think Global Health. 2023. https://www.thinkglobalhealth.org/article/rise-and-fall-seguro-popular-mexicos-health-care-odyssey. Accessed 9 Jan 2025.

[62] Pines N, Bala M, Gross I, Ohana-Sarna-Cahan L, Shpigel R, Nama A, et al. Changes in pediatric major trauma epidemiology, injury patterns, and outcome during COVID-19–associated lockdown. Trauma. 2023;25:62–6. 10.1177/14604086211045359. 36883119 10.1177/14604086211045359 PMC9982404

[63] Maigrot J-LA, Zhou G, Koroukian SM, Weiss AJ, Gillinov AM, Bakaeen F, et al. Nationwide analysis of case volume and outcomes in cardiac surgery during the COVID-19 pandemic. JTCVS Open. 2024;19:200–9. 10.1016/j.xjon.2024.02.024. 39015470 10.1016/j.xjon.2024.02.024 PMC11247236

[64] Servin-Rojas M, Olivas-Martinez A, Ramirez Del Val F, Torres-Gomez A, Navarro-Vargas L, García-Juárez I. Transplant trends in Mexico during the COVID-19 pandemic: Disparities within healthcare sectors. Am J Transpl. 2021;21:4052–60. 10.1111/ajt.16801. 10.1111/ajt.16801 PMC8441748 34387936

[65] González-Calatayud M, Zacarías-Ezzat JR. Surgical impact of the severe acute respiratory syndrome coronavirus 2 pandemic in a third-level hospital in Mexico City. Rev Med Hosp Gen Mex. 2020;83:182–8. 10.24875/hgmx.20000068.

[66] Rabbone I, Tagliaferri F, Carboni E, Crotti B, Ruggiero J, Monzani A, et al. Changing Admission Patterns in Pediatric Emergency Departments during the COVID-19 Pandemic in Italy Were Due to Reductions in Inappropriate Accesses. Child (Basel). 2021;8:962. 10.3390/children8110962. 10.3390/children8110962 PMC8620376 34828676

[67] Goldman RD, Grafstein E, Barclay N, Irvine MA, Portales-Casamar E. Paediatric patients seen in 18 emergency departments during the COVID-19 pandemic. Emerg Med J. 2020;37:773–7. 10.1136/emermed-2020-210273. 33127743 10.1136/emermed-2020-210273

[68] Cuenca A, Coy A, Gutiérrez N, Santos MP, Bustos JD, Morales AM, et al. Change in pediatric trauma-related visits in a tertiary hospital in Colombia during coronavirus disease 2019 lockdown. Pediatr Emerg Med J. 2023;10:17–22.

[69] Nachon-Acosta A, Martinez-Mier G, Flores-Gamboa V, Avila-Mercado O, Garcia IM, Yoldi-Aguirre C, et al. Surgical Outcomes During COVID-19 Pandemic. Arch Med Res. 2021;52:434–42. 10.1016/j.arcmed.2021.01.003. 33618912 10.1016/j.arcmed.2021.01.003 PMC7825836

[70] La Jornada. Los fines de semana el IMSS hará cirugías programadas. 2021. https://www.jornada.com.mx/notas/2021/03/15/politica/los-fines-de-semana-el-imss-hara-cirugias-programadas/. Accessed 28 June 2025.

[71] Johnson & Johnson. LATAM Tracker - Return to surgery. 2020. https://www.jnjmedtech.com/sites/default/files/user_uploaded_assets/pdf_assets/2021-01/LA-001_Elective%20Surgeries%20Report%20-%20May%2021st%202020.pdf. Accessed 28 June 2025.

[72] Campos LN, Bryce-Alberti M, Gerk A, Hill SK, Calderon C, Zaigham M, et al. Examining the surgical backlog due to COVID-19 in Latin America and the Caribbean: insights from a scoping review. Lancet Reg Health Am. 2024;40:100908. 10.1016/j.lana.2024.100908. 39493415 10.1016/j.lana.2024.100908 PMC11530758

[73] Mendivelso Duarte FO, Rodríguez Bedoya M, Barrios Parra AJ. [Recommendations for reopening elective surgery services during the SARS-CoV-2 pandemic]. Rev Panam Salud Publica. 2020;44:e114. 10.26633/RPSP.2020.114. 32952535 10.26633/RPSP.2020.114 PMC7491862

[74] Frio GS, Russo LX, de Albuquerque CP, da Mota LMH, Barros-Areal AF, Oliveira APRA, et al. The disruption of elective procedures due to COVID-19 in Brazil in 2020. Sci Rep. 2022;12:10942. 10.1038/s41598-022-13746-5. 35768482 10.1038/s41598-022-13746-5 PMC9243075

[75] Ng-Kamstra JS, Arya S, Greenberg SLM, Kotagal M, Arsenault C, Ljungman D, et al. Perioperative mortality rates in low-income and middle-income countries: a systematic review and meta-analysis. BMJ Glob Health. 2018;3:e000810. 10.1136/bmjgh-2018-000810. 29989045 10.1136/bmjgh-2018-000810 PMC6035511

[76] Livingston MH, DCruz J, Pemberton J, Ozgediz D, Poenaru D. Mortality of pediatric surgical conditions in low and middle income countries in Africa. J Pediatr Surg. 2015;50:760–4. 10.1016/j.jpedsurg.2015.02.031. 25783373 10.1016/j.jpedsurg.2015.02.031

[77] Nwankwo EP, Onyejesi DC, Chukwu IS, Modekwe VI, Nwangwu EI, Ezomike UO, et al. Pediatric Perioperative Mortality in Southeastern (SE) Nigeria—A Multicenter, Prospective Study. Niger J Clin Pract. 2025;28:225. 10.4103/njcp.njcp_695_24. 40326905 10.4103/njcp.njcp_695_24

[78] Murni IK, Djer MM, Yanuarso PB, Putra ST, Advani N, Rachmat J, et al. Outcome of pediatric cardiac surgery and predictors of major complication in a developing country. Ann Pediatr Cardiol. 2019;12:38–44. 10.4103/apc.APC_146_17. 30745768 10.4103/apc.APC_146_17 PMC6343386

[79] Hu R, Zhu H, Qiu L, Hong H, Xu Z, Zhang H, et al. Association Between Preoperative Factors and In-hospital Mortality in Neonates After Cardiac Surgery in China. Front Pediatr. 2021;9:670197. 10.3389/fped.2021.670197. 34422714 10.3389/fped.2021.670197 PMC8374182

[80] Cervantes-Salazar J, Calderón-Colmenero J, Ramírez-Marroquín S, Palacios-Macedo A, Bolio Cerdán A, Vizcaíno Alarcón A, et al. Mexican registry of pediatric cardiac surgery. First report. Bol Med Hosp Infant Mex. 2014;71:286–91. 10.1016/j.bmhimx.2014.07.003. 29421617 10.1016/j.bmhimx.2014.07.003

[81] Berger JT, Holubkov R, Reeder R, Wessel DL, Meert K, Berg RA, et al. Morbidity and mortality prediction in pediatric heart surgery: Physiological profiles and surgical complexity. J Thorac Cardiovasc Surg. 2017;154:620–e6286. 10.1016/j.jtcvs.2017.01.050. 28274558 10.1016/j.jtcvs.2017.01.050 PMC5519426

[82] Zheng G, Wu J, Chen P, Hu Y, Zhang H, Wang J, et al. Characteristics of in-hospital mortality of congenital heart disease (CHD) after surgical treatment in children from 2005 to 2017: a single-center experience. BMC Pediatr. 2021;21:521. 10.1186/s12887-021-02935-2. 34814864 10.1186/s12887-021-02935-2 PMC8609813

[83] Calderón-Colmenero J, Cervantes-Salazar J, Curi-Curi P, Ramírez-Marroquín S. Congenital heart disease in Mexico: advances of the regionalization project. World J Pediatr Congenit Heart Surg. 2013;4:165–71. 10.1177/2150135113477868. 23799729 10.1177/2150135113477868

[84] Armenta-Paulino N, Wehrmeister FC, Arroyave L, Barros AJD, Victora CG. Ethnic inequalities in health intervention coverage among Mexican women at the individual and municipality levels. eClinicalMedicine. 2021;43:101228. 10.1016/j.eclinm.2021.101228. 34927037 10.1016/j.eclinm.2021.101228 PMC8649218

[85] Leyva-Flores R, Servan-Mori E, Infante-Xibille C, Pelcastre-Villafuerte BE, Gonzalez T. Primary Health Care Utilization by the Mexican Indigenous Population: The Role of the Seguro Popular in Socially Inequitable Contexts. PLoS ONE. 2014;9:e102781. 10.1371/journal.pone.0102781. 25099399 10.1371/journal.pone.0102781 PMC4123888

[86] Ramos LGF, Navarro SM. Feb. The Health of Indigenous Populations in Mexico: Disencounters | ReVista. https://revista.drclas.harvard.edu/the-health-of-indigenous-populations-in-mexico-disencounters/. Accessed 5 2026.

[87] Prado BH, Angulo EMR, Palmisano EB, Rodríguez RO, Baranda RJO, Pecha MGA, et al. Factors associated with delays in the search for care in under-5 deaths in Yucatán, Mexico. Salud Publica Mex. 2021;63:498–508. 10.21149/12216. 34098595 10.21149/12216 PMC9201850

[88] Leyva-Flores R, Infante-Xibille C, Gutiérrez JP, Quintino-Pérez F. Persisting health and health access inequalities in Mexican indigenous population, 2006–2012. Salud Pública de México. 2013;55:S123–8. 24626687

[89] Salas-Ortiz A. Socioeconomic Inequalities and Ethnic Discrimination in COVID-19 Outcomes: the Case of Mexico. J Racial Ethn Health Disparities. 2024;11:900–12. 10.1007/s40615-023-01571-z. 37041406 10.1007/s40615-023-01571-z PMC10089566

[90] Gray M, Williams K, Oster RT, Bruno G, Cooper A, Healy C, et al. Indigenous identity identification in administrative health care data globally: A scoping review. J Health Serv Res Policy. 2024;29:210–21. 10.1177/13558196231219955. 38099443 10.1177/13558196231219955 PMC11151709

[91] Gartner DR, Maples C, Nash M, Howard-Bobiwash H. Misracialization of Indigenous people in population health and mortality studies: a scoping review to establish promising practices. Epidemiol Rev. 2023;45:63–81. 10.1093/epirev/mxad001. 37022309 10.1093/epirev/mxad001 PMC10748801

[92] Duarte-Raya F, Baeza-Zarco FJ. Incidencia y factores de riesgo asociados a infección nosocomial en cardiocirugía pediátrica. Rev Med Inst Mex Seguro Soc. 2016;54:182–9. 26960046

[93] Garcia Garcia JA, Del Valle DD, Wurdeman T, Ashi K, Bistre Varon J, Angulo-Lozano JC, et al. Burn injuries among paediatric patients treated at Mexican public hospitals: a retrospective cohort analysis of nationwide hospitalisation data. BMJ Glob Health. 2025;10:e017915. 10.1136/bmjgh-2024-017915. 40132808 10.1136/bmjgh-2024-017915 PMC11938246

[94] Sonpar A, Hundal CO, Totté JEE, Wang J, Klein SD, Twyman A, et al. Multimodal strategies for the implementation of infection prevention and control interventions. Clin Microbiol Infect. 2025;31:948–57. 10.1016/j.cmi.2025.01.011. 39863071 10.1016/j.cmi.2025.01.011

[95] Shamah-Levy T, Gaona-Pineda EB, Cuevas-Nasu L, Morales-Ruan C, Valenzuela-Bravo DG, Humaran IM-G, et al. Prevalencias de sobrepeso y obesidad en población escolar y adolescente de México. Ensanut Continua 2020–2022. Salud Publica Mex. 2023;65:s218–24. 10.21149/14762. 38060970 10.21149/14762

[96] Rivera JÁ, de Cossío TG, Pedraza LS, Aburto TC, Sánchez TG, Martorell R. Childhood and adolescent overweight and obesity in Latin America: a systematic review. Lancet Diabetes Endocrinol. 2014;2:321–32. 10.1016/S2213-8587(13)70173-6. 24703050 10.1016/S2213-8587(13)70173-6

[97] Turnbull B, Gordon SF, Martínez-Andrade GO, González-Unzaga M. Childhood obesity in Mexico: A critical analysis of the environmental factors, behaviours and discourses contributing to the epidemic. Health Psychol Open. 2019;6:2055102919849406. 10.1177/2055102919849406. 31205736 10.1177/2055102919849406 PMC6537260

[98] Brero M, Meyer CL, Jackson-Morris A, Spencer G, Ludwig-Borycz E, Wu D, et al. Investment case for the prevention and reduction of childhood and adolescent overweight and obesity in Mexico. Obes Rev. 2023;24:e13595. 10.1111/obr.13595. 37464960 10.1111/obr.13595

[99] Crowe H. If Chile can stop children eating junk food, why can't Britain? The Times. 2025.

[100] Paul ME, Wallace JG, Coakley BA. An Assessment of the Relationship Between BMI and Children Undergoing Surgical Procedures: A Retrospective Study. Child Obes. 2023;19:249–57. 10.1089/chi.2022.0065. 35776521 10.1089/chi.2022.0065 PMC10398724

[101] Stey AM, Moss LR, Kraemer K, Cohen ME, Ko CY, Hall BL. The Importance of Extreme Weight Percentile in Postoperative Morbidity in Children. J Am Coll Surg. 2014;218:988–96. 10.1016/j.jamcollsurg.2013.12.051. 24680569 10.1016/j.jamcollsurg.2013.12.051

[102] Rivero-Moreno Y, Garcia A, Rivas-Perez M, Coa-Bracho J, Salcedo Y, Gonzalez-Quinde G, et al. Effect of Obesity on Surgical Outcomes and Complication Rates in Pediatric Patients: A Comprehensive Systematic Review and Meta-Analysis. Cureus. 2024;16:e54470. 10.7759/cureus.54470. 38510855 10.7759/cureus.54470 PMC10953840

[103] Servan-Mori E, Torres-Pereda P, Orozco E, Sosa-Rubí SG. An explanatory analysis of economic and health inequality changes among Mexican indigenous people, 2000–2010. Int J Equity Health. 2014;13:21. 10.1186/1475-9276-13-21. 24576113 10.1186/1475-9276-13-21 PMC3996059

