# Supplementary material for: Epidemiology of children and adolescents undergoing surgery at Mexican public hospitals: a retrospective registry-based analysis from 2010 to 2022
Source: BMC Pediatr. 2026 May 21;26:553. doi: 10.1186/s12887-026-07011-1 (PMC13251272; doi:10.1186/s12887-026-07011-1)
Supplement: Supplementary file 1 — Supplementary Material 1. [file 12887_2026_7011_MOESM1_ESM.docx]

# Supplementary

## Supplementary Table 1: Flowchart of inclusion and exclusion criteria for pediatric surgical procedures in Mexico, 2010–2022 (final analysis population: 752,654)


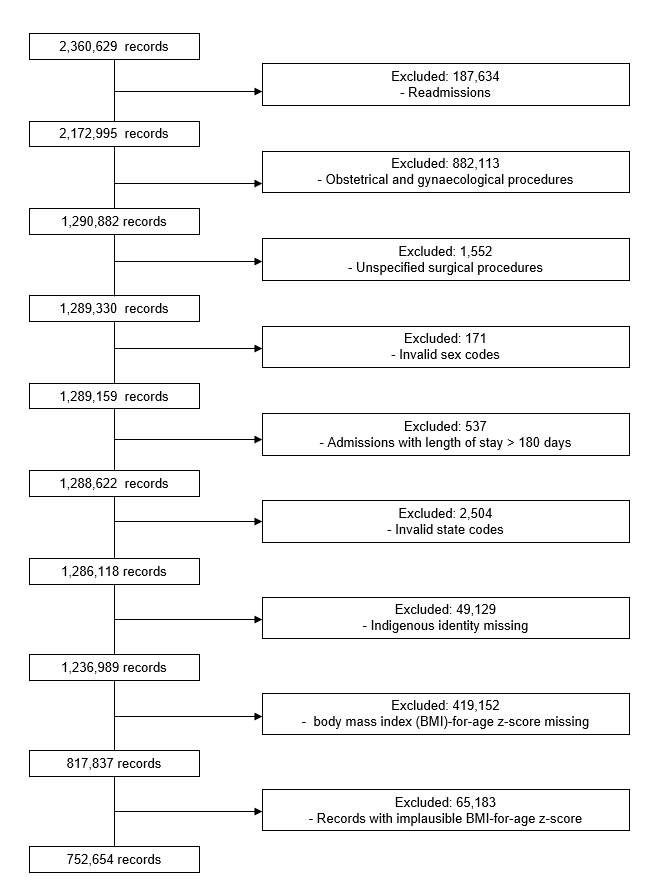


## Supplementary Table 2: In-hospital mortality among patients excluded from the analysis population due to missing BMI-for-age z-scores (n = 1,236,989)

|  |  | **Total (n=1,236,989)** |
| --- | --- | --- |
| Mortality | No | 1,224,624 (99.0%) |
|  | Yes | 12,365 ( 1.0%) |

## Supplementary Table 3: Surgical procedures among males (n=437,888) stratified by age group in Mexico from 2010-2022

| **Surgical Procedure** | **Under 5 years** | **%** | **5-9 years** | **%** | **10-14 years** | **%** | **15-17 years** | **%** | **Total (100.0%)** |
| --- | --- | --- | --- | --- | --- | --- | --- | --- | --- |
| **Cardiothoracic surgery** | 8,392 | 72.8 | 695 | 6.0 | 927 | 8.0 | 1,521 | 13.2 | 11,535 |
| **Endocrine surgery** | 260 | 35.5 | 213 | 29.1 | 161 | 22.0 | 99 | 13.5 | 733 |
| **General surgery** | 46,248 | 24.1 | 46,245 | 24.1 | 61,876 | 32.3 | 37,374 | 19.5 | 191,743 |
| **Multiple procedures** | 5,982 | 34.5 | 3,384 | 19.5 | 4,325 | 25.0 | 3,624 | 20.9 | 17,315 |
| **Neurosurgery** | 4,835 | 48.8 | 1,423 | 14.4 | 1,991 | 20.1 | 1,653 | 16.7 | 9,902 |
| **Ophthalmology** | 2,889 | 42.7 | 1,713 | 25.3 | 1,389 | 20.5 | 769 | 11.4 | 6,760 |
| **Orthopedic surgery** | 18,295 | 15.1 | 33,120 | 27.3 | 44,718 | 36.9 | 25,035 | 20.7 | 121,168 |
| **Otolaryngology** | 13,423 | 46.0 | 7,683 | 26.3 | 5,124 | 17.6 | 2,952 | 10.1 | 29,182 |
| **Urology** | 22,374 | 45.2 | 12,359 | 24.9 | 10,802 | 21.8 | 4,015 | 8.1 | 49,550 |
| **Total** | 122,698 | 28.0 | 106,835 | 24.4 | 131,313 | 30.0 | 77,042 | 17.6 | 437,888 |

%: percentage

## Supplementary Table 4: Surgical procedures among females (n=314,766) stratified by age group in Mexico from 2010-2022

| **Surgical Procedure** | **Under 5 years** | **%** | **5-9 years** | **%** | **10-14 years** | **%** | **15-17 years** | **%** | **Total (100.0%)** |
| --- | --- | --- | --- | --- | --- | --- | --- | --- | --- |
| **Cardiothoracic surgery** | 7,040 | 75.9 | 636 | 6.9 | 764 | 8.2 | 839 | 9.0 | 9,279 |
| **Endocrine surgery** | 230 | 24.4 | 197 | 20.9 | 236 | 25.0 | 280 | 29.7 | 943 |
| **General surgery** | 32,374 | 20.1 | 34,447 | 21.4 | 48,420 | 30.1 | 45,451 | 28.3 | 160,692 |
| **Multiple procedures** | 2,673 | 5.2 | 1,436 | 2.8 | 5,283 | 10.3 | 41,727 | 81.6 | 51,119 |
| **Neurosurgery** | 3,931 | 57.7 | 971 | 14.3 | 1,093 | 16.0 | 815 | 12.0 | 6,810 |
| **Ophthalmology** | 2,544 | 39.6 | 1,462 | 22.8 | 1,433 | 22.3 | 988 | 15.4 | 6,427 |
| **Orthopedic surgery** | 14,341 | 26.2 | 16,118 | 29.5 | 15,411 | 28.2 | 8,863 | 16.2 | 54,733 |
| **Otolaryngology** | 8,765 | 39.1 | 6,134 | 27.3 | 4,553 | 20.3 | 2,988 | 13.3 | 22,440 |
| **Urology** | 1,210 | 52.1 | 323 | 13.9 | 320 | 13.8 | 470 | 20.2 | 2,323 |
| **Total** | 73,108 | 23.2 | 61,724 | 19.6 | 77,513 | 24.6 | 102,421 | 32.5 | 314,766 |

%: percentage

## **Supplementary Table 5:** Age-standardized incidence rates (ASIR) of **surgical procedures in children and adolescents (per 100,000) stratified by Mexican states and year (n=**752,654)

|  | **2010** | **2011** | **2012** | **2013** | **2014** | **2015** | **2016** | **2017** | **2018** | **2019** | **2020** | **2021** | **2022** | **Total** |
| --- | --- | --- | --- | --- | --- | --- | --- | --- | --- | --- | --- | --- | --- | --- |
| **Aguascalientes** | 12.3 | 9.1 | 21.1 | 6.8 | 9.3 | 19.5 | 30.7 | 14.8 | 20.3 | 81.8 | 97.0 | 113.9 | 99.3 | 41.4 |
| **Baja California** | 48.3 | 90.9 | 116.7 | 122.3 | 135.1 | 111.0 | 81.6 | 87.5 | 68.1 | 72.4 | 46.8 | 109.2 | 112.6 | 92.4 |
| **Baja California Sur** | 183.4 | 166.3 | 219.4 | 242.5 | 278.1 | 274.0 | 301.5 | 219.3 | 232.2 | 226.6 | 117.9 | 122.1 | 193.1 | 213.3 |
| **Campeche** | 131.2 | 163.7 | 243.3 | 191.5 | 179.3 | 144.8 | 222.0 | 242.6 | 199.7 | 173.6 | 119.0 | 153.6 | 155.1 | 178.8 |
| **Coahuila de Zaragoza** | 66.5 | 72.5 | 80.3 | 75.2 | 87.6 | 90.7 | 99.0 | 104.6 | 97.6 | 98.1 | 59.3 | 92.0 | 120.7 | 88.2 |
| **Colima** | 235.9 | 286.4 | 308.8 | 320.3 | 335.3 | 323.7 | 262.0 | 219.0 | 261.0 | 251.3 | 136.6 | 118.5 | 129.3 | 246.6 |
| **Chiapas** | 75.9 | 99.0 | 100.2 | 110.6 | 104.9 | 111.9 | 100.8 | 90.7 | 82.4 | 82.4 | 66.5 | 58.4 | 56.7 | 88.5 |
| **Chihuahua** | 153.4 | 165.8 | 165.6 | 161.8 | 84.4 | 141.6 | 113.2 | 106.3 | 103.5 | 89.9 | 54.3 | 80.0 | 77.4 | 115.2 |
| **Ciudad de México** | 69.8 | 65.3 | 68.4 | 90.9 | 112.1 | 119.3 | 119.2 | 82.2 | 85.0 | 100.3 | 66.7 | 66.2 | 53.2 | 84.8 |
| **Durango** | 254.9 | 272.8 | 313.9 | 291.3 | 286.7 | 298.4 | 289.2 | 257.5 | 248.3 | 224.3 | 144.4 | 212.6 | 220.9 | 255.5 |
| **Guanajuato** | 294.6 | 341.0 | 349.8 | 338.6 | 368.1 | 375.2 | 380.7 | 353.8 | 334.0 | 356.9 | 261.6 | 265.9 | 313.9 | 333.8 |
| **Guerrero** | 157.4 | 161.4 | 208.4 | 222.4 | 242.5 | 250.2 | 257.1 | 222.8 | 218.1 | 185.6 | 120.7 | 126.1 | 139.5 | 194.1 |
| **Hidalgo** | 203.8 | 213.5 | 174.5 | 169.6 | 179.2 | 172.9 | 179.5 | 140.4 | 176.3 | 177.2 | 104.7 | 109.6 | 140.7 | 164.9 |
| **Jalisco** | 124.8 | 135.5 | 137.5 | 147.1 | 135.8 | 126.3 | 120.8 | 109.8 | 107.6 | 115.3 | 62.4 | 65.0 | 73.2 | 112.6 |
| **México** | 202.4 | 207.0 | 204.6 | 231.9 | 222.2 | 230.2 | 231.7 | 223.8 | 167.5 | 175.9 | 141.1 | 129.8 | 128.8 | 192.6 |
| **Michoacán** | 191.7 | 215.4 | 202.2 | 205.7 | 229.6 | 238.1 | 287.0 | 217.4 | 191.6 | 129.7 | 67.1 | 75.7 | 85.3 | 181.2 |
| **Morelos** | 111.6 | 121.2 | 123.1 | 151.3 | 152.3 | 144.3 | 140.1 | 122.2 | 115.2 | 121.3 | 79.8 | 90.2 | 102.0 | 121.3 |
| **Nayarit** | 312.9 | 274.0 | 272.6 | 244.6 | 207.2 | 252.8 | 258.7 | 204.2 | 232.0 | 224.5 | 126.3 | 148.5 | 246.3 | 231.0 |
| **Nuevo León** | 30.9 | 30.1 | 27.4 | 22.8 | 19.6 | 17.1 | 17.9 | 15.4 | 18.1 | 9.6 | 10.2 | 11.4 | 12.6 | 18.5 |
| **Oaxaca** | 201.4 | 217.0 | 230.1 | 230.2 | 220.4 | 228.1 | 206.4 | 170.6 | 181.1 | 133.0 | 97.1 | 85.4 | 171.1 | 183.4 |
| **Puebla** | 126.8 | 152.9 | 160.3 | 147.3 | 185.7 | 217.3 | 207.5 | 188.1 | 170.8 | 177.7 | 112.0 | 136.0 | 175.3 | 165.9 |
| **Querétaro de Arteaga** | 28.3 | 41.7 | 42.9 | 48.5 | 49.4 | 81.6 | 68.6 | 89.5 | 128.7 | 103.1 | 96.6 | 168.8 | 176.5 | 87.4 |
| **Quintana Roo** | 94.7 | 67.8 | 87.1 | 96.2 | 76.0 | 92.9 | 67.1 | 104.3 | 156.5 | 132.3 | 78.4 | 94.2 | 111.4 | 97.6 |
| **San Luis Potosí** | 27.8 | 33.9 | 68.5 | 96.2 | 55.0 | 111.4 | 106.0 | 87.6 | 90.6 | 81.2 | 54.2 | 55.8 | 72.5 | 72.2 |
| **Sinaloa** | 236.6 | 233.5 | 199.6 | 151.6 | 177.4 | 198.1 | 193.5 | 176.6 | 180.7 | 168.6 | 86.6 | 137.4 | 197.8 | 180.5 |
| **Sonora** | 78.0 | 51.4 | 57.5 | 50.0 | 51.8 | 54.5 | 56.4 | 54.2 | 57.7 | 63.3 | 39.1 | 38.5 | 37.6 | 53.2 |
| **Tabasco** | 355.3 | 380.4 | 358.7 | 340.5 | 350.6 | 333.3 | 342.0 | 463.0 | 228.2 | 278.4 | 178.3 | 245.9 | 277.4 | 319.3 |
| **Tamaulipas** | 88.0 | 96.0 | 113.3 | 114.5 | 117.4 | 130.1 | 112.2 | 107.4 | 121.1 | 106.0 | 63.9 | 81.8 | 91.3 | 103.6 |
| **Tlaxcala** | 373.8 | 429.2 | 441.9 | 353.0 | 363.7 | 351.8 | 344.4 | 268.7 | 299.6 | 247.0 | 112.7 | 212.5 | 299.3 | 315.9 |
| **Veracruz** | 81.3 | 79.4 | 69.7 | 65.6 | 73.5 | 73.6 | 75.7 | 101.1 | 93.9 | 96.5 | 59.6 | 71.7 | 89.3 | 79.1 |
| **Yucatán** | 44.4 | 62.8 | 85.7 | 111.9 | 129.5 | 135.7 | 135.9 | 134.8 | 115.4 | 73.4 | 75.1 | 105.2 | 115.0 | 101.9 |
| **Zacatecas** | 66.9 | 50.8 | 63.1 | 60.3 | 113.6 | 142.0 | 145.7 | 147.7 | 162.7 | 157.5 | 78.7 | 115.1 | 156.7 | 112.1 |
| **Total** | 135.5 | 148.1 | 154.1 | 156.6 | 160.3 | 165.9 | 161.3 | 150.7 | 142.7 | 139.7 | 91.4 | 106.7 | 127.0 | 141.8 |

## Supplementary Table 6: Average monthly surgical procedures in Mexico from 2010-2022 **(n=**752,654) stratified by sex (males=437,888; females=314,766)

| **Year** | **Male** | **Female** | **Overall** |
| --- | --- | --- | --- |
| **2010** | 2,719.9 | 1,991.5 | 4,711.4 |
| **2011** | 2,965.0 | 2,177.4 | 5,142.4 |
| **2012** | 3,104.9 | 2,231.8 | 5,336.7 |
| **2013** | 3,133.2 | 2,275.9 | 5,409.1 |
| **2014** | 3,205.7 | 2,314.3 | 5,520.0 |
| **2015** | 3,307.2 | 2,382.2 | 5,689.4 |
| **2016** | 3,178.7 | 2,331.9 | 5,510.6 |
| **2017** | 2,951.1 | 2,166.3 | 5,117.4 |
| **2018** | 2,776.8 | 2,043.5 | 4,820.3 |
| **2019** | 2,727.8 | 1,960.9 | 4,688.7 |
| **2020** | 1,783.3 | 1,268.0 | 3,051.3 |
| **2021** | 2,117.8 | 1,419.6 | 3,537.4 |
| **2022** | 2,519.3 | 1,667.2 | 4,186.5 |

## Supplementary Table 7: Interrupted time series analysis (ITS) with Poisson regression results for overall surgical procedures in Mexico from 2010-2022 **(n=**752,654) stratified by sex (males=437,888; females=314,766)

| **Model** | **Variable** | **β** | **exp(β)** | **95% Confidence Interval** |
| --- | --- | --- | --- | --- |
| **Male** | Pre-pandemic trend (linear) | 0.0092915 | 1.01 | 1.01, 1.01 |
|  | Pre-pandemic trend (quadratic) | -0.0000830 | 1.00 | 1.00, 1.00 |
|  | April 2020 level change | -0.4693803 | 0.63 | 0.57, 0.68 |
|  | Pandemic trend change (linear) | -0.0074106 | 0.99 | 0.98, 1.01 |
|  | Pandemic trend change (quadratic) | 0.0009276 | 1.00 | 1.00, 1.00 |
| **Female** | Pre-pandemic trend (linear) | 0.0091707 | 1.01 | 1.01, 1.01 |
|  | Pre-pandemic trend (quadratic) | -0.0000822 | 1.00 | 1.00, 1.00 |
|  | April 2020 level change | -0.4358838 | 0.65 | 0.60, 0.70 |
|  | Pandemic trend change (linear) | -0.0159107 | 0.98 | 0.97, 1.00 |
|  | Pandemic trend change (quadratic) | 0.0011075 | 1.00 | 1.00, 1.00 |
| **Overall** | Pre-pandemic trend (linear) | 0.0093183 | 1.01 | 1.01, 1.01 |
|  | Pre-pandemic trend (quadratic) | -0.0000835 | 1.00 | 1.00, 1.00 |
|  | April 2020 level change | -0.4392418 | 0.65 | 0.59, 0.70 |
|  | Pandemic trend change (linear) | -0.0089877 | 0.99 | 0.98, 1.01 |
|  | Pandemic trend change (quadratic) | 0.0009314 | 1.00 | 1.00, 1.00 |

β: beta coefficient, exp(β): exponentiated beta coefficient

## Supplementary Table 8: Interrupted time series analysis (ITS) with Poisson regression stratified by surgical specialty (n=752,654) in Mexico from 2010-2022

| **Surgical Specialty** | **Variable** | **β** | **exp(β)** | **95% Confidence Interval** |
| --- | --- | --- | --- | --- |
| **Cardiothoracic surgery** | Pre-pandemic trend (linear) | 0.0154596 | 1.02 | 1.01, 1.02 |
|  | Pre-pandemic trend (quadratic) | -0.0001038 | 1.00 | 1.00, 1.00 |
|  | April 2020 level change | 0.0734384 | 1.08 | 1.00, 1.16 |
|  | Pandemic trend (linear) | 0.0421199 | 1.04 | 1.03, 1.06 |
|  | Pandemic trend (quadratic) | -0.0001632 | 1.00 | 1.00, 1.00 |
| **Endocrine surgery** | Pre-pandemic trend (linear) | 0.0225951 | 1.02 | 1.01, 1.03 |
|  | Pre-pandemic trend (quadratic) | -0.0002067 | 1.00 | 1.00, 1.00 |
|  | April 2020 level change | -1.0690040 | 0.34 | 0.27, 0.43 |
|  | Pandemic trend (linear) | -0.0292355 | 0.97 | 0.92, 1.02 |
|  | Pandemic trend (quadratic) | 0.0025698 | 1.00 | 1.00, 1.00 |
| **General surgery** | Pre-pandemic trend (linear) | 0.0088404 | 1.01 | 1.01, 1.01 |
|  | Pre-pandemic trend (quadratic) | -0.0000835 | 1.00 | 1.00, 1.00 |
|  | April 2020 level change | -0.3741872 | 0.69 | 0.63, 0.75 |
|  | Pandemic trend (linear) | -0.0089002 | 0.99 | 0.97, 1.01 |
|  | Pandemic trend (quadratic) | 0.0009345 | 1.00 | 1.00, 1.00 |
| **Multiple procedures** | Pre-pandemic trend (linear) | 0.0107516 | 1.01 | 1.01-1.01 |
|  | Pre-pandemic trend (quadratic) | -0.0000660 | 1.00 | 1.00-1.00 |
|  | April 2020 level change | -0.3187938 | 0.73 | 0.68-0.77 |
|  | Pandemic trend (linear) | -0.0142273 | 0.99 | 0.97-1.00 |
|  | Pandemic trend (quadratic) | 0.0007928 | 1.00 | 1.00-1.00 |
| **Neurosurgery** | Pre-pandemic trend (linear) | 0.0013508 | 1.00 | 1.00-1.00 |
|  | Pre-pandemic trend (quadratic) | -0.0000186 | 1.00 | 1.00-1.00 |
|  | April 2020 level change | -0.3565982 | 0.70 | 0.64-0.77 |
|  | Pandemic trend (linear) | 0.0272902 | 1.03 | 1.01-1.05 |
|  | Pandemic trend (quadratic) | -0.0006942 | 1.00 | 1.00-1.00 |
| **Ophthalmology** | Pre-pandemic trend (linear) | 0.0077209 | 1.01 | 1.00-1.01 |
|  | Pre-pandemic trend (quadratic) | -0.0000992 | 1.00 | 1.00-1.00 |
|  | April 2020 level change | -1.0059250 | 0.37 | 0.30-0.45 |
|  | Pandemic trend (linear) | -0.0560593 | 0.94 | 0.91-0.99 |
|  | Pandemic trend (quadratic) | 0.0027700 | 1.00 | 1.00-1.00 |
| **Orthopedic surgery** | Pre-pandemic trend (linear) | 0.0085327 | 1.01 | 1.01-1.01 |
|  | Pre-pandemic trend (quadratic) | -0.0000752 | 1.00 | 1.00-1.00 |
|  | April 2020 level change | -0.3429694 | 0.71 | 0.66-0.77 |
|  | Pandemic trend (linear) | 0.0005468 | 1.00 | 0.98-1.02 |
|  | Pandemic trend (quadratic) | 0.0004699 | 1.00 | 1.00-1.00 |
| **Otolaryngology** | Pre-pandemic trend (linear) | 0.0079513 | 1.01 | 1.00-1.01 |
|  | Pre-pandemic trend (quadratic) | -0.0000894 | 1.00 | 1.00-1.00 |
|  | April 2020 level change | -2.6654730 | 0.07 | 0.06-0.08 |
|  | Pandemic trend (linear) | -0.0856077 | 0.92 | 0.88-0.96 |
|  | Pandemic trend (quadratic) | 0.0034061 | 1.00 | 1.00-1.01 |
| **Urology** | Pre-pandemic trend (linear) | 0.0170052 | 1.02 | 1.01-1.02 |
|  | Pre-pandemic trend (quadratic) | -0.0001468 | 1.00 | 1.00-1.00 |
|  | April 2020 level change | -1.1313420 | 0.32 | 0.28-0.37 |
|  | Pandemic trend (linear) | -0.0219894 | 0.98 | 0.95-1.01 |

β: beta coefficient, exp(β): exponentiated beta coefficient

## Supplementary Table 9: Average monthly surgical procedures stratified by surgical specialty in Mexico from 2010-2022 **(n=**752,654)

| **Surgical specialty** | **Year** | **Average monthly procedures** |
| --- | --- | --- |
| **Cardiothoracic surgery** | 2010 | 90.8 |
|  | 2011 | 110.3 |
|  | 2012 | 103.3 |
|  | 2013 | 112.2 |
|  | 2014 | 144.8 |
|  | 2015 | 152.1 |
|  | 2016 | 140.5 |
|  | 2017 | 134.1 |
|  | 2018 | 129.5 |
|  | 2019 | 121.4 |
|  | 2020 | 119.6 |
|  | 2021 | 168.9 |
|  | 2022 | 207.1 |
| **Endocrine surgery** | 2010 | 11.2 |
|  | 2011 | 11.8 |
|  | 2012 | 12.2 |
|  | 2013 | 12.3 |
|  | 2014 | 17.0 |
|  | 2015 | 19.2 |
|  | 2016 | 12.6 |
|  | 2017 | 12.3 |
|  | 2018 | 8.3 |
|  | 2019 | 9.2 |
|  | 2020 | 3.9 |
|  | 2021 | 4.3 |
|  | 2022 | 6.2 |
| **General surgery** | 2010 | 2256.8 |
|  | 2011 | 2463.7 |
|  | 2012 | 2587.4 |
|  | 2013 | 2577.0 |
|  | 2014 | 2602.6 |
|  | 2015 | 2653.9 |
|  | 2016 | 2615.0 |
|  | 2017 | 2352.6 |
|  | 2018 | 2178.3 |
|  | 2019 | 2155.8 |
|  | 2020 | 1423.6 |
|  | 2021 | 1601.2 |
|  | 2022 | 1901.8 |
| **Multiple procedures** | 2010 | 373.7 |
|  | 2011 | 402.4 |
|  | 2012 | 356.3 |
|  | 2013 | 436.1 |
|  | 2014 | 447.6 |
|  | 2015 | 485.1 |
|  | 2016 | 517.6 |
|  | 2017 | 514.4 |
|  | 2018 | 510.3 |
|  | 2019 | 473.0 |
|  | 2020 | 360.0 |
|  | 2021 | 385.4 |
|  | 2022 | 441.0 |
| **Neurosurgery** | 2010 | 107.5 |
|  | 2011 | 114.1 |
|  | 2012 | 108.2 |
|  | 2013 | 110.2 |
|  | 2014 | 106.0 |
|  | 2015 | 108.4 |
|  | 2016 | 109.4 |
|  | 2017 | 110.3 |
|  | 2018 | 97.2 |
|  | 2019 | 94.6 |
|  | 2020 | 111.3 |
|  | 2021 | 104.5 |
|  | 2022 | 111.1 |
| **Ophthalmology** | 2010 | 102.9 |
|  | 2011 | 106.0 |
|  | 2012 | 113.3 |
|  | 2013 | 108.6 |
|  | 2014 | 109.3 |
|  | 2015 | 101.6 |
|  | 2016 | 93.8 |
|  | 2017 | 87.6 |
|  | 2018 | 77.3 |
|  | 2019 | 78.1 |
|  | 2020 | 26.5 |
|  | 2021 | 39.3 |
|  | 2022 | 54.5 |
| **Orthopedic Surgery** | 2010 | 1086.3 |
|  | 2011 | 1180.7 |
|  | 2012 | 1255.2 |
|  | 2013 | 1233.4 |
|  | 2014 | 1259.4 |
|  | 2015 | 1316.8 |
|  | 2016 | 1249.9 |
|  | 2017 | 1194.7 |
|  | 2018 | 1145.5 |
|  | 2019 | 1102.1 |
|  | 2020 | 766.1 |
|  | 2021 | 907.2 |
|  | 2022 | 961.3 |
| **Otolaryngology** | 2010 | 383.4 |
|  | 2011 | 408.8 |
|  | 2012 | 413.8 |
|  | 2013 | 418.1 |
|  | 2014 | 425.2 |
|  | 2015 | 421.8 |
|  | 2016 | 365.8 |
|  | 2017 | 346.2 |
|  | 2018 | 346.8 |
|  | 2019 | 339.2 |
|  | 2020 | 101.7 |
|  | 2021 | 131.3 |
|  | 2022 | 199.9 |
| **Urology** | 2010 | 298.8 |
|  | 2011 | 344.8 |
|  | 2012 | 387.1 |
|  | 2013 | 401.3 |
|  | 2014 | 408.1 |
|  | 2015 | 430.6 |
|  | 2016 | 405.9 |
|  | 2017 | 365.3 |
|  | 2018 | 327.2 |
|  | 2019 | 315.4 |
|  | 2020 | 139.3 |
|  | 2021 | 195.3 |
|  | 2022 | 303.7 |
